# A cross-scale multimodal framework identifies clinically actionable immunotherapy biomarkers in melanoma through bulk to single-cell and spatial transcriptomics integration

**DOI:** 10.1186/s40246-026-00919-w

**Published:** 2026-03-16

**Authors:** Wuda Huoshen, Kun Yuan, Junkai Xiong, Yilong Lin, Wenjie Yu, Yun Xie, Qian Yuan, Xinyue Zhang, Changqing Dong, Chen Sun, Sha Yi

**Affiliations:** 1 Department of Dermatology, Chengdu Integrated TCM & Western Medicine Hospital, Chengdu, Sichuan China; 2 School of Stomatology, Southwest Medical University, Luzhou, Sichuan China; 3 Clinical Medical College, Southwest Medical University, Luzhou, Sichuan China; 4 Depeartment of Breast Surgery, the First Affiliated Hospital of Xiamen University, School of Medicine, Xiamen University, Xiamen, China; 5 Cancer Center of Beijing Tsinghua Changgung Hospital, School of Clinical Medicine, Tsinghua Medicine, Tsinghua University, Beijing, China; 6 College of Stomatology, Chongqing Medical University, Chongqing, China; 7 Chongqing Key Laboratory of Oral Diseases, Chongqing, China; 8 Chongqing Municipal Key Laboratory of Oral Biomedical Engineering of Higher Education, Chongqing, China; 9 Department of Critical Care Medicine, Shanghai General Hospital, Shanghai Jiao Tong University School of Medicine, Songjiang, Shanghai 201600 People’s Republic of China; 10 iSoftStone Information Technology (Group) Co., Ltd., Nanjing, Jiangsu China; 11 Department of Endodontics, The Affiliated Stomatological Hospital, Southwest Medical University, Luzhou, Sichuan China; 12 Department of Nephrology, The Second Hospital of Jilin University, National Key Laboratory of Diabetes, Changchun, China; 13 Department of Periodontics and Oral Mucosal Diseases, The Affiliated Stomatology Hospital, Southwest Medical University, Luzhou, Sichuan China

**Keywords:** Cutaneous melanoma, Multi-omics, Prognostic model, Single-cell transcriptomics, Spatial transcriptomics, TCGA-SKCM cohort, Transcriptome-wide association study, Tumor microenvironment characteristics, Therapeutic targets

## Abstract

**Background:**

Melanoma, a type of skin cancer that can spread to other parts of the body, currently lacks highly precise individualized treatment options.

**Methods:**

We performed multi-omics integration on The Cancer Genome Atlas Skin Cutaneous Melanoma (TCGA-SKCM) cohort to identify melanoma molecular subtypes. The identified genes were validated in independent meta cohorts from GEO, followed by transcriptome-wide association study (TWAS) validation using Genotype-Tissue Expression (GTEx) and UK Biobank datasets. Additionally, we analyzed machine learning-driven signature (CMLS) development, tumor microenvironment characteristics, immunotherapy response, and potential therapeutic targets. Finally, single-cell and spatial transcriptomics provided further biological insights and the pathomechanisms.

**Result:**

Our study identified two distinct molecular subtypes of SKCM using multimodal data integration with the MOVICS package: Cancer Subtype 1 (CS1) and CS2. CS2 showed a better prognosis and was enriched in immune-suppressive pathways such as WNT–β signaling, while CS1 exhibited higher activation of the PI3K pathway and DNA repair mechanisms, along with greater tumor invasiveness. TWAS analysis results combined the findings from TCGA-SKCM and the meta-cohort, identifying six significant prognostic-related genes (SPRGs). The CMLS prognostic model, based on SPRGs (*CAP2*, *SELL*, *and LAPTM5* as risk factors and *GZMA*, *FCER1G*, and *LYZ* as protective factors), stratified patients into high-group (poorer survival) and low-risk groups. Single-cell and spatial transcriptomic analyses further validated CMLS prognostic results, highlighting distinct tumor microenvironment interactions and progression trajectories.

**Conclusion:**

Identifications of molecular subtypes and CMLS represent a valuable tool for early prediction of patient prognosis and for screening potential candidates likely to benefit from immunotherapy, with broad implications for clinical practice foundation for personalized therapies.

**Supplementary Information:**

The online version contains supplementary material available at 10.1186/s40246-026-00919-w.

## Introduction

Melanoma is a malignant tumor from melanocytes, primarily found in the skin [1]. Skin cutaneous melanoma (SKCM), which accounts for over 90% of cases, predominantly affects individuals of White populations, following by Black or Brown skin color and east Asian populations [1]. The pathogenesis of this disease is largely attributed to exposure to ultraviolet radiation (UVR) [2]. Epidemiologic surveys have shown that the incidence of melanoma is on the rise globally, especially in areas where fair-skinned people are overexposed to the sunlight, and that the incidence in Europe has continued to increase in recent years in all age groups and is expected to continue in the coming decades [3].

SKCM is primarily managed with wide excision [1], while non-surgical approaches include the use of topical imiquimod 5% cream and radiation therapy [4–6]. For individuals diagnosed with high-risk resectable melanoma, current standards recommend adjuvant treatment with either anti-death protein 1(anti-PD-1) checkpoint inhibitors or BRAF-targeted therapies, both of which have demonstrated significant efficacy in decreasing tumor recurrence rates and prolonging distant metastasis-free survival outcomes [1, 5, 7]. In the treatment of advanced melanoma, tumor-infiltrating lymphocyte (TIL) therapy, targeted therapies, and checkpoint immunotherapies are commonly utilized [8–10]. However, strategies focused on the individual treatment of SKCM have achieved only limited success so far. Immunotherapy has garnered significant research attention and shown positive results in some patients. Nonetheless, many patients continue to see limited clinical benefit from this treatment [11, 12]. This may be because most cancer patients do not respond to Immune checkpoint inhibitors (ICIs), and a significant number of those who do develop either innate or acquired resistance, limiting the clinical effectiveness of the treatment [13]. Considering the high costs and potential for severe side effects of immunotherapy, there is a clear need to utilize large-scale multi-omics data and advanced machine learning algorithms to identify biomarkers that can help predict outcomes and guide immunotherapy management in SKCM patients.

In this study, we performed a subtype analysis using an integrative multi-omics approach combining mRNA, long non-coding RNA (lncRNA), and microRNA (miRNA) expression profiles, genomic mutations, and epigenomic DNA methylation data to develop a prognostic model, further validated through single-cell transcriptomics and spatial transcriptomics. Initially, a multi-omics integration analysis was conducted on The Cancer Genome Atlas Skin Cutaneous Melanoma (TCGA-SKCM) cohort to identify molecular subtypes of melanoma. The genes identified in the training cohorts were validated in independent meta-validation cohorts from GEO. Subsequently, transcriptome-wide association study (TWAS) analysis was conducted as an additional validation step using independent cohorts from the Genotype-Tissue Expression (GTEx) project and the UK Biobank. Furthermore, we conducted comprehensive analyses to gain important molecular insights into melanoma, focusing on the consensus machine learning-driven signature (CMLS), tumor microenvironment characteristics, immunotherapy response, and potential therapeutic targets. Finally, single-cell and spatial transcriptomics (ST) analyses were used to validate these findings.

## Materials and methods

### Study design

**Fig. 1:**
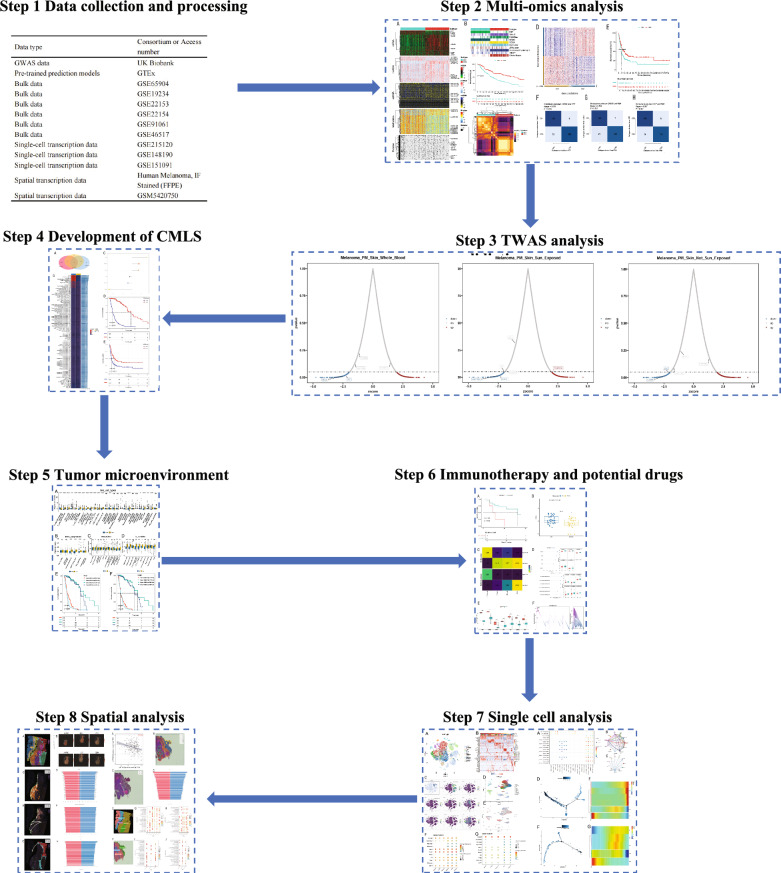
The overall study design and workflow, illustrating the multi-omics integration process, molecular subtyping of melanoma, immune microenvironment characterization, and validation through external datasets, single-cell, and spatial transcriptomics analyses

The flowchart of our study was presented in Fig. 1. First, a multi-omics integration analysis was performed on the TCGA-SKCM cohort to identify molecular subtypes of metastatic melanoma. The genes identified in the training cohorts were then validated using independent meta-GEO validation cohorts from GEO, followed by TWAS analysis using independent validation cohorts from GTEx and the UK Biobank. Additionally, comprehensive analyses were conducted on the development of CMLS, tumor microenvironment characteristics, immunotherapy response, and potential therapeutic targets. Finally, the findings were further validated through single-cell transcriptomics and spatial transcriptomics to enhance biological insights.

### TCGA preprocessing of multi-omics and multi-center cohort data for SKCM

We collected multi-omics data of metastatic melanoma from the TCGA-SKCM cohort (https://portal.gdc.cancer.gov), which includes transcriptomic expression profiles of mRNA and lncRNA, DNA methylation data, somatic mutation data, and clinical information. The transcriptomic profiles of mRNA and lncRNA were obtained using the TCGAbiolinks R package (version = 2.30.4) [14], while the mature miRNA IDs were recorded using the mirbaseVersions.db R package (version = 3.18) [15]. Somatic mutations were retrieved using TCGAbiolinks and processed with maftools. DNA methylation profiles and clinical data were downloaded from UCSC Xena (https://xenabrowser.net/). We performed several quality control steps: (1) Removing clinical samples with missing values (denoted as 'NA') or infinite values; (2) Retaining only protein-coding genes in the mRNA dataset; (3) Calculating the mean TPM (transcripts per million) for each gene, lncRNA, and miRNA; and (4) Applying a log transformation for normalization. Somatic mutations were counted using maftools. For RNA sequencing data, TPM normalization was used, as it is comparable with microarray-based gene expression profiles and enhances sample comparability.

In addition to TCGA-SKCM, we included 11 external SKCM datasets for validation: meta-GEO cohort (combined with six bulk RNA-seq datasets cohorts)(GSE65904, GSE19234, GSE22153, GSE22154, GSE91061, GSE46517; available at http://www.ncbi.nlm.nih.gov/geo), three single-cell RNA-seq datasets (GSE215120, GSE148190, GSE151091), and two spatial transcriptomics datasets (ST) including the GSM5420750 sample from GSE179572 (https://www.ncbi.nlm.nih.gov/geo/query/acc.cgi?acc=GSM5420750) and Human Melanoma, IF Stained from 10 × Genomics (Mastering Biology to Advance Human Health—10 × Genomics).

After downloading microarray-based datasets, expression matrices, and clinical information, we excluded samples without survival status and survival time. Background correction, log2 transformation, and quantile normalization were performed. To merge different datasets, the “ComBat” function from the “sva” package was applied to adjust for batch effects and technical biases. To validate the effectiveness of the data merging, we performed Principal Component Analysis (PCA) on the merged samples both before and after the merging process.

### Multi-omics consensus integration analysis

To perform an effective integrative analysis, we first matched five-dimensional omics data using sample IDs (n = 337). TPM expression data were log2-transformed. For DNA methylation data, we selected probes from CpG islands of promoters. For gene mutation matrices, mutations were defined when genes contained any nonsynonymous variants involving frameshift insertions or deletions, in-frame insertions or deletions, nonsense or missense mutations, splicing site mutations, and mutations at translation initiation sites. In this study, we used the “getElites” function from the Multi-Omics Integration and Expansion for Cancer Subtypes (MOVICS) package (version = 0.99.17) to filter gene features [16]. For continuous variables (mRNA, lncRNA, miRNA, and methylation), we set the “method” parameter of the “getElites” function to “mad” to select the top 1,500 genes with the highest variation. We then set the “method” parameter to “cox” and integrated clinical data to identify prognostically relevant genes (*P* < 0.05) as part of an upstream dimensionality-reduction step recommended in the official MOVICS workflow, prior to unsupervised integrative clustering. For binary variable gene mutation data, we further screened genes based on mutation frequency, setting the “method” parameter to “freq” to identify the top 5% most frequent mutated genes.

After preliminary feature selection, we determined the optimal number of clusters for our study. It is well known that the best number of clusters should be small enough to reduce interference but large enough to retain essential information. Therefore, we employed the “getClustNum” function from the MOVICS package, which integrates clustering prediction indices (CPI), Gap-statistics, and Silhouette scores to estimate the number of subgroups. Drawing on prior knowledge from studies of urothelial carcinoma, we ultimately decided to classify SKCM into two subtypes and applied the “getMOIC” function for result analysis. Additionally, based on differential expression analysis between the subtypes, we selected 100 subtype-specific upregulated genes as classifiers. Ten clustering algorithms (CIMLR, ConsensusClustering, SNF, iClusterBayes, PINSPlus, moCluster, NEMO, IntNMF, COCA, and LRA) were used as inputs for the “methodslist” parameter and used the default parameters provided by the MOVICS package. We obtained clustering results for each method, and based on these results, we integrated the clustering outcomes using the “getConsensusMOIC” function, improving the robustness of clustering by applying the concept of consensus clustering. The “distance” and “linkage” parameters were set to “Euclidean,” and “average”, respectively. The final clustering results were derived through this integrated process. The multi-omics clustering and integration were conducted strictly following the official MOVICS workflow and recommended parameters, as detailed in the published MOVICS vignette (https://xlucpu.github.io/MOVICS/MOVICS-VIGNETTE.html#Section.4).

### Subtype-specific molecular features and stability

Using Gene Set Variation Analysis (GSVA) [17], we calculated enrichment scores for multi-therapy-related features, including cancer development-related features, molecular subtypes associated with SKCM, and features related to targeted therapy and radiotherapy. We then incorporated 28 induced/repressed target-related transcription factors (TFs) and 71 candidate regulators related to chromatin remodeling in carcinogenesis, as identified by Lu et al. [18]. We compared the distribution of immune checkpoints across these subgroups, and the ESTIMATE R package was used to predict immune/stromal scores for tumor tissues. Tumor-infiltrating lymphocyte DNA methylation (MeTIL) scores were calculated according to published protocols (reference). For subtype stability, we firstly validated the clustering results using subtype-specific biomarkers from validation cohorts and then compared the consensus clustering with the consistency of NTP and PAM classifiers.

### TWAS analysis validation

We conducted a TWAS analysis using the PrediXcan method to identify the genes which screened by the TCGA-SKCM and meta-GEO [19], which utilizes elastic net regression to determine the optimal hyperparameters. The analysis was based on RNA sequencing data from the GTEx project (V8) and employed pre-trained prediction models available on Zenodo (https://zenodo.org/records/3518299).

For the genome-wide association studies (GWAS) data, we used melanoma skin data from the UK Biobank, which includes approximately 500,000 participants aged 40–69 years at recruitment [20]. The case definition for melanoma was based on ICD10 diagnoses, including inpatient, outpatient, and cause-of-death records. In total, 2,824 cases and 453,524 controls were included in the analysis (https://www.ebi.ac.uk/gwas/studies/GCST90041829).

### Development of a consensus machine learning-driven prognostic feature

To enhance the comparability across different cohorts, we preprocessed all data by performing Z-score normalization. To assess the relationship between CMLS, immunotherapy, and prognosis, we selected the TCGA cohort with relatively complete treatment information as the training set, and other cohorts as validation sets. Considering the small sample sizes in some cohorts, we merged four cohorts into a cohort named meta-GEO. To build a CMLS model with high accuracy and generalizability, we integrated ten machine learning algorithms, including random survival forest (RSF), elastic network (Enet), Lasso, Ridge, stepwise Cox, CoxBoost, partial least squares regression for Cox (plsRcox), supervised principal components (SuperPC), generalized boosted regression modeling (GBM), and survival support vector machine (survival-SVM) [21]. Notably, algorithms like stepwise Cox, RSF, CoxBoost, and Lasso have feature selection capabilities. During the model-building phase, we used the TCGA cohort as the training set for preliminary model construction. The analysis was conducted using the online platform "Bioinformatics Sprout" (101 Algorithm Combinations for Prognostic Model Construction—Bioinformatics Sprout, a specialized platform for bioinformatics analysis).

### CMLS development pipeline

We performed univariate Cox analysis on the TCGA-SKCM and meta-GEO SKCM cohorts. Genes with a *p*-value < 0.05 and consistent hazard ratio (HR) directions across all cohorts were considered as significant prognostic-related genes (SPRGs). We applied ten machine learning algorithms, using 101 combinations to construct the most predictive CMLS with optimal C-index performance. After developing the model on the training set, we further tested it across all validation cohorts. We calculated the average C-index for each model, with the highest value indicating the best-performing model.

### Prognostic value, immunological characterization, and immunotherapy response analysis

Based on the CMLS model, we scored each sample in the training and validation sets, classifying them into high CMLS and low CMLS groups based on their scores. Kaplan–Meier survival curves were used to assess the prognostic significance of CMLS. The distribution of patients within the CMLS-High and CMLS-Low subgroups in Additional file 1: Table S1.

Using the IOBR package [22], we collected several previously published features related to tumor microenvironment (TME) cell types, immunotherapy response, immune suppression, and immune evasion. We applied a unified method to compute enrichment scores for each sample, providing a comprehensive analysis of immune differences between high and low CMLS patients. We compared the differences in Tumor Mutation Burden (TMB) and CD8 + T-cell distribution, and combined CMLS to reclassify patients. For immunotherapy response, we firstly incorporated the GSE91061 cohort, plotted survival curves of high and low CMLS patients after anti-PD1 treatment, and utilized subtype mapping to predict immunotherapy responses [23].

### Computerized analysis to identify potential therapeutic drugs for high CMLS patients

We analyzed the activation status of carcinogenic pathways between high and low CMLS patients using GSEA (Gene Set Enrichment Analysis) (62). Extracted data for human cancer cell lines (CCL) were obtained from the Broad Institute’s Cancer Cell Line Encyclopedia (CCLE). Drug sensitivity data for CCLs were sourced from CTRP v.2.0 (https://portals.broadinstitute.org/ctrp) and the PRISM Repurposing dataset (https://depmap.org/portal/prism/). Dose–response curve areas under the curve (AUC) were used as measures of drug sensitivity.

### Single-cell level analysis of CMLS

Firstly, we downloaded single-cell transcriptomic data (GSE215120, GSE148190, GSE151091) from the public repository. From these datasets, we selected four skin malignant melanoma samples from GSE215120, and six lymphatic samples with triple files from GSE148190 as tumor samples, and a merged dataset of 22 normal nevus samples classified as normal samples. The data was read using the rhdf5 (R package, version 2.46.1) and Seurat (R package, version 4.3.0.1) packages, and merged using the Merge function.

To process the unique molecular identifier (UMI) count matrix, Seurat was applied, and cells with UMI/gene counts exceeding the average limits (± two times the median absolute deviation) were filtered, if the UMI/gene count distribution follows a Gaussian distribution. The parameters were set to minGene = 500, maxGene = 5000, and pctMT = 20 to remove low-quality cells. Following this, library size normalization was performed using the NormalizeData() function in Seurat to obtain normalized counts with log transformation was applied. The top 2000 highly variable genes were identified using the FindVariableFeatures() function, following a previously described method.

Principal components (PCs) were computed based on the expression profiles of the top 2000 highly variable genes. Cell clustering was performed using the FindNeighbors() and FindClusters() functions in Seurat. For visualization, the RunUMAP() function was used to apply a 2D Uniform Manifold Approximation and Projection (UMAP) algorithm, and the results were visualized with the DimPlot() function. Marker genes for each cluster were identified using the FindAllMarkers() function in Seurat identifying positive markers for a given cluster compared with all remaining clusters. To avoid any influence from the "Unknown" types, we excluded them from further analysis.

### Trajectory analysis

Developmental pseudo-time analysis was performed using the package (version 2.30.1) to infer the developmental trajectory of cells [24]. To reduce the number of cells for analysis, a random subset of half of the cells from the Seurat object was used. The RNA count data was extracted from the Seurat object and converted into a sparse matrix format to ensure compatibility with Monocle. Cell and gene metadata were then extracted from the Seurat object and used to create AnnotatedDataFrame objects that construct a CellDataSet object in Monocle. Size factors were estimated to normalize gene expression across cells, and gene dispersion values were calculated to facilitate downstream differential expression analysis. Genes with low expression were filtered out, and a set of highly variable genes was selected for trajectory inference based on differential gene expression analysis with a q-value threshold of < 0.01. These genes were used to order cells along the developmental trajectory, and a pseudo-time trajectory was constructed using Monocle’s trajectory inference functions.

### Cell–cell communication analysis with CellChat

Cell–cell communication analysis was performed using the R package (version 1.1.3) to evaluate potential intercellular communication [25]. The analysis began by setting the Seurat object’s identity to cell type information, which was then used for downstream analyses. A CellChat object was created from the Seurat object using the createCellChat() function. The human CellChat database was assigned to the CellChat object by specifying CellChatDB.human as the reference.

The analysis pipeline involved several steps: Firstly, the data was subsetted to remove low-quality cells or genes using the subsetData() function; Secondly, the identifyOverExpressedGenes() function was applied to identify genes overexpressed in different cell populations; Thirdly, the identifyOverExpressedInteractions() function was used to detect potential intercellular interactions based on the overexpressed genes; Fourthly, the computeCommunProb() function was used to calculate the likelihood of cell-to-cell communication, and interactions involving fewer than 50 cells were filtered out using the filterCommunication() function; Fifthly, the computeCommunProbPathway() function was employed to compute communication probabilities based on signaling pathways, and finally, the interaction network was aggregated using the aggregateNet() function, consolidating similar interactions into a single representation. The centrality of nodes within the network was calculated using the netAnalysis_computeCentrality() function to assess the most influential cells in the communication network.

### Scissors algorithm for identifying phenotype-associated cells

The Scissors algorithm is an innovative method for analyzing single-cell data, leveraging bulk phenotypes to identify subpopulations of cells that are highly correlated with these phenotypes from single-cell sequencing data [26]. For our analysis, high CMLS was chosen as the phenotype in the logistic regression model. The parameter alpha was set to 0.5, and the family was specified as binomial to identify the most relevant hypoxic subtypes.

### ST data analysis

ST data were processed and visualized using the Seurat R package. The Load10X_Spatial() function was employed to load the processed ST data, and the initial data were visualized using VlnPlot() and SpatialFeaturePlot() functions, which provided insights into the distribution of spatially expressed features, such as "nCount_Spatial" and "percent.mt". Mitochondrial and ribosomal genes were filtered out using regular expressions to exclude these genes from further analysis. Additionally, genes expressed in fewer than 10 spots were excluded to ensure high-quality data.

The ST data were then standardized using the SCTransform method to mitigate technical noise. Dimensionality reduction was performed via PCA, and clustering was applied based on the first 10 principal components, followed by visualization using UMAP. For metabolic analysis, the “scMetabolism” package (version = 0.2.1) was utilized to evaluate metabolic activity within the ST dataset [27]. The AUCell method was applied to assess metabolic pathways from the KEGG database, and the DotPlot.metabolism() function was used to visualize the expression of key metabolic pathways across different cell types. This integrated approach allowed for both spatial mapping and metabolic profiling, providing deeper insights for further interpretation.

Additionally, we directly visualized the relevant genes in the obtained Human Melanoma, IF Stained (FFPE) dataset (Mastering Biology to Advance Human Health—10 × Genomics) using the official Loupe Browser software. “Tumor nest”, “melanoma cells”, and “tumor margin” regions were defined in the spatial dataset, this process was conducted by experienced clinical dermatologists and senior oncology researchers to ensure pathological accuracy.

### Statistical analysis

For comparisons between two groups, normally distributed variables were analyzed using the unpaired Student’s t-test, while non-normally distributed variables were analyzed using the Wilcoxon rank-sum test. For comparisons among more than two groups, parametric and non-parametric variables were tested using one-way ANOVA and the Kruskal–Wallis test, respectively. Two-sided Fisher’s exact test was used for contingency tables. The critical value of CMLS scores was determined using the “surv-cutpoint” function from the survminer package (Version = 0.5.0). We repeatedly tested all potential cutoff points to identify the highest-ranking statistic and then classified the CMLS scores using a two-category method. Based on the maximum selected log-rank statistic, patients in each cohort were classified into high and low groups to reduce compound batch effects, consistent with previous studies [28–30]. TWAS analysis was performed in Linux operating system. Differential expression analysis was performed using the limma package (3.58.1) [31], and multi-omics clustering was conducted through the MOVICS package [16]. All statistical analyses were performed in R (version 4.3.2).

## Results

### Multimodal consensus and prognosis-related molecular subtypes of SKCM

**Fig. 2:**
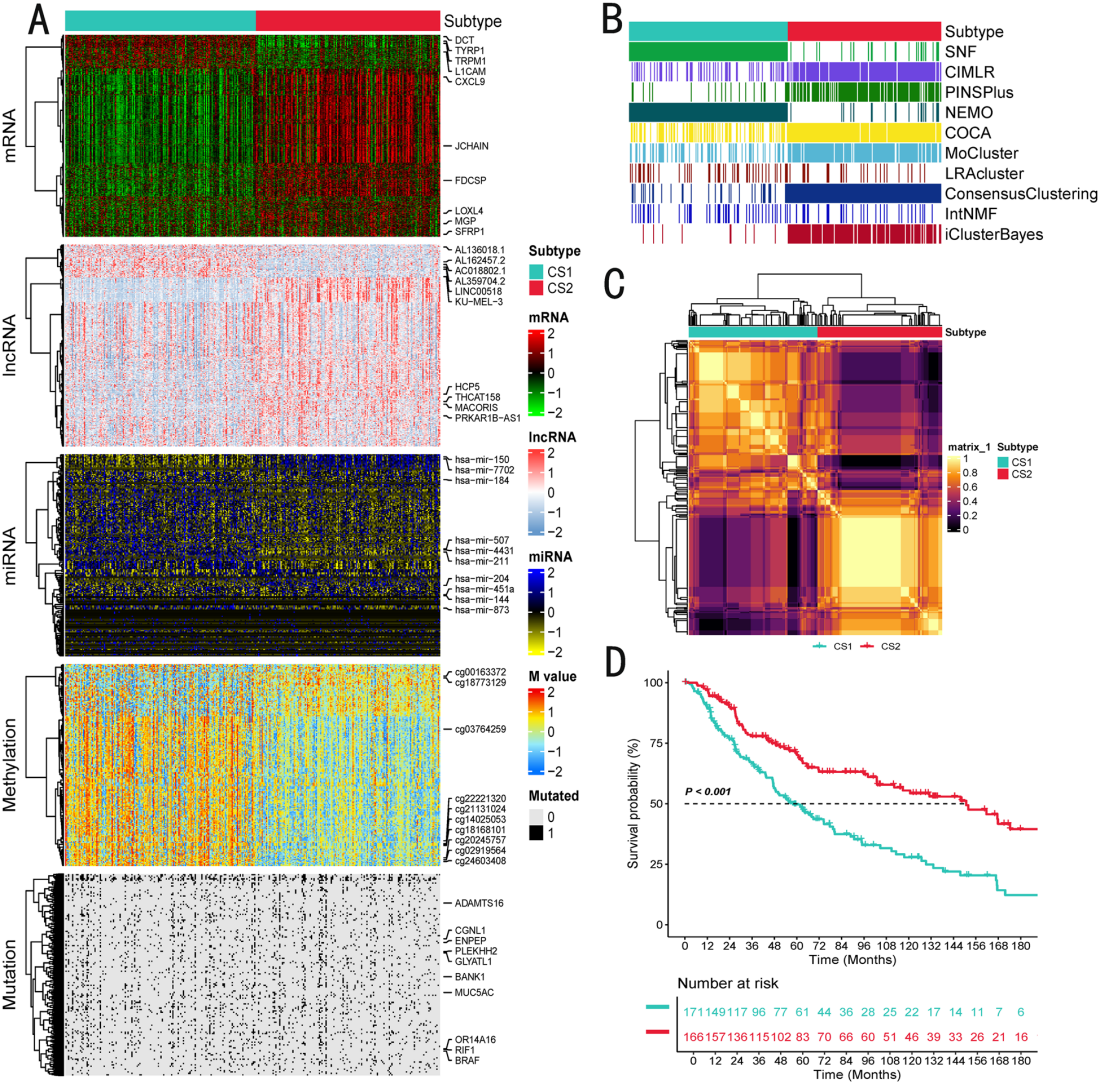
The multiomics integrative consensus subtypes of SKCM. **A** Comprehensive heatmap of consensus ensemble subtypes, including mRNA, lncRNA, miRNA, DNA CpG methylation site, and mutant gene. **B** Clustering of SKCM patients through 10 cutting-edge multiomics clustering methods. **C** Consensus clustering matrix for three novel prognostic subtypes based on the 10 algorithms. **D** Different survival outcomes among the three subtypes

We analyzed the five datasets (mRNA, lncRNA, and miRNA expression profiles, genomic mutations, and epigenomic DNA methylation data), using the MOVICS package, and 10 multimodal integrated clustering algorithms, in a total of 337 melanoma samples with matched multi-omics profiles, identifying two distinct subtypes: Cancer Subtype 1 (CS1) and CS2 (Fig. 2A–C). We then determined the optimal number of subtypes by referencing the Cluster Prediction Index, Gap-statistics, silhouette scores, and previous research experience (Additional file 2: Figure S1-S2). Our classification system is strongly associated with overall survival (OS) (*P* < 0.001) (Fig. 2D). Notably, Cancer Subtype 2 (CS2) exhibited better survival outcomes. A summary of clinical covariates across CS1/CS2 subtypes and high/low CMLS groups is provided (Additional file 1: Table S3), and multivariable Cox analysis adjusting for age and stage confirms the independent prognostic value of both CS1/CS2 and CMLS (Additional file 1: Table S4, Additional file 2: Figure S3). We validated the effectiveness of data merging by performing principal component analysis (PCA) on the samples before and after merging, demonstrating improved sample integration after the merging process (Additional file 2: Figure S4 and S5).

### Integration and consensus-based molecular subtyping of SKCM

**Fig. 3:**
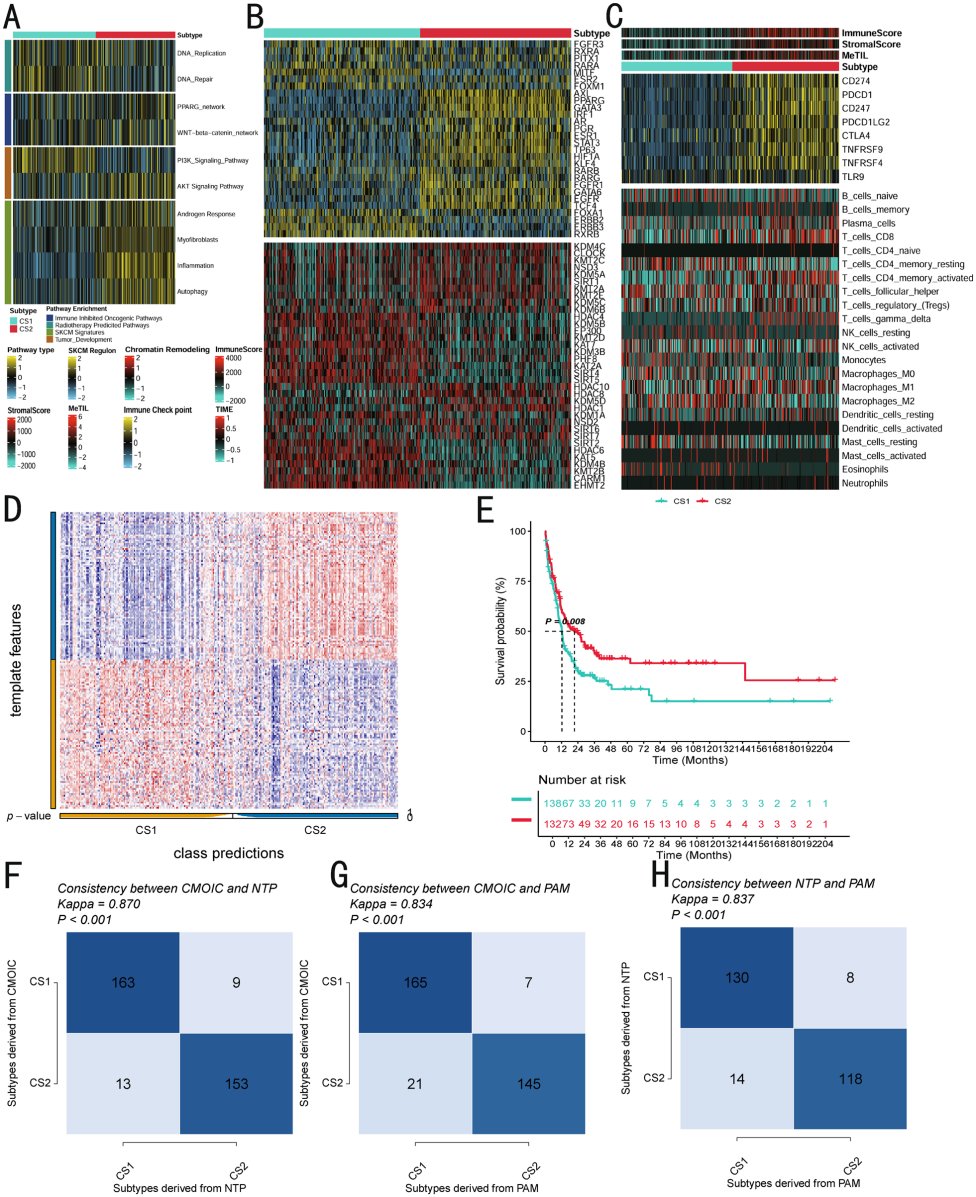
Molecular landscape and validation of SKCM CSs. **A** The enrichment of three subtypes for different treatment-related signatures and bladder cancer-related signatures. **B** Regulon activity profiles for 28 TFs (top) and potential regulators associated with chromatin remodeling (bottom) of two subtypes. **C** Immune profiles in the TCGA-SKCM cohort. The top annotation of the heatmap shows the immune enrichment score, stromal enrichment score, and DNA methylation of tumor-infiltrating lymphocytes. The top panel shows the expression of canonical immune checkpoint genes, and the bottom panel shows the enrichment levels of 24 TME-related immune cells. **D** Validation of SKCM CSs in the nearest template of the meta-GEO SKCM cohort. **E** Survival analysis of SKCM CSs in the meta-GEO SKCM cohort. **F** The consistency of NTP with CMOIC in the META-SKCM cohort. **G** The consistency of CMOIC with PAM in the META-SKCM cohort. **H** The consistency of NTP with PAM in the meta-GEO SKCM cohort

Currently, most SKCM molecular subtypes are categorized based on molecular expression levels, which are associated with specific biological functions. Therefore, we also aimed to explore the distinct molecular characteristics of these two subtypes. We utilized GSVA to measure the enrichment of various molecular features in the samples (Fig. 3A). In CS2, significant enrichment was observed in the AKT signaling pathway, inflammatory signaling pathways, and autophagy pathways. In contrast, CS1 showed upregulated expression in the PI3K pathway and DNA repair and replication, while also displaying a significant downregulation in fibrosis. This fibrotic degradation may suggest that CS1 has greater invasiveness during tumor progression. Furthermore, these two subtypes showed considerable differences in their response to specific treatments and pathways such as CS2 was relatively enriched in immune-suppressive carcinogenic pathways, like WNT-β signaling, while CS1 may be more likely to benefit from radiotherapy or targeted therapies.

To further investigate transcriptomic differences, we analyzed potential regulatory factors related to cancer chromatin remodeling and 28 transcription factors (TFs) associated with SKCM (Fig. 3B). The activity of these regulatory factors was closely linked to the subtypes, confirming the biological relevance of the subtypes. *MITF* and *AXL*, known to be associated with poor prognosis in SKCM, were significantly overexpressed in CS1, while *TP63*, a tumor-suppressor gene, exhibited higher expression in CS2. The regulatory activity profiles related to cancerous chromatin remodeling further emphasized the differential regulatory patterns between the subtypes, suggesting that epigenetically driven transcriptional networks may be critical factors distinguishing these molecular subtypes.

Given the key role of the immune system in tumor initiation and progression, we quantified the activity of immune checkpoints and the infiltration levels of microenvironment cells (Fig. 3C). We found that immune checkpoints were significantly activated in CS2, with high expression of CD274 promoting T lymphocyte-mediated control of tumor cell growth. TNFRSF9, via the p38MAPK/PAX6 signaling pathway, inhibited cancer progression, enhanced T-cell function, and reduced exhaustion phenotypes. Regarding immune cell infiltration, CS2 showed relatively higher expression of gamma delta T cells, suggesting that CS2 may have an advantage in early recognition and rapid response to malignant cells. To further validate the stability of these subtypes, we combined four cohorts (GSE65904, GSE19234, GSE22153, GSE22154) after batch correction, naming the resulting meta-cohort. The closest template prediction (NTP) classified each sample in the external cohort into one of the identified subtypes (Fig. 3D). Consistent with this, CS2 exhibited better prognosis in the validation cohort (*P* = 0.008) (Fig. 3E), and the stability of the subtypes was confirmed through CMOIC, NTP, and PAM algorithms (Fig. 3F–H).

### TWAS analysis validation and construction of CMLS

**Fig. 4:**
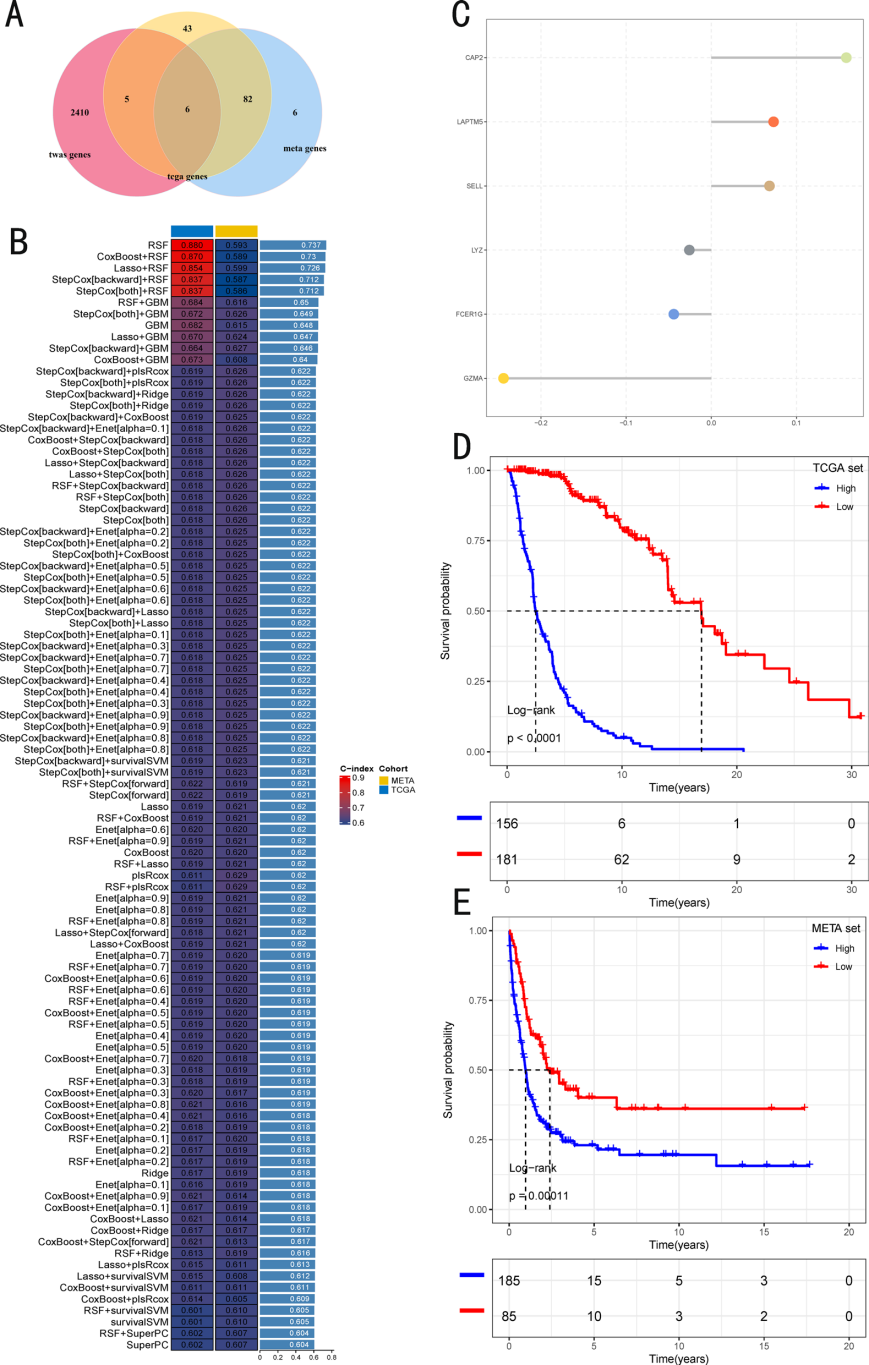
The generation and prognostic value of CMLS. **A** The intersection of genes from the TWAS, TCGA, and meta-GEO SKCM cohorts were taken to obtain SPRGs. **B** Through a comprehensive computational framework, a combination of 101 machine learning algorithms was generated. The C-index of each model was calculated through the TCGA-SKCM and meta-GEO SKCM cohorts and sorted by the average C-index of the validation set. **C** The hub gene selected through the CoxBoost algorithm. **D**, **E** Survival analysis of SKCM patients with high CMLS and low CMLS in META-SKCM cohort and TCGA-SKCM cohort

In sun-protected skin tissues, *LYZ* and *GZMA* were validated and found to be associated with melanoma (Additional file 2: Figure S6), while in sun-exposed skin tissues, *LAPTM5*, *CAP2*, and *FCER1G* were identified and linked to melanoma (Additional file 2: Figure S7). In whole blood, *SELL* and *LYZ* were validated and shown to be associated with melanoma (Additional file 2: Figure S8). Although the specific genes identified varied across tissues, the majority are immune-related, and the observed tissue-specific associations likely reflect context-dependent gene regulation across different tissue and cellular environments.We performed univariate Cox regression analysis and TWAS analysis validation to screen 6 SPRGs (*CAP2*, *SELL*, *LAPTM5*, *GZMA*, *FCER1G*, and *LYZ*) whose expression was significantly correlated with OS from the TCGA and meta-GEO cohorts (Fig. 4A). Subsequently, these SPRGs were integrated into a framework for constructing the CMLS. Using TCGA as the training set and meta-GEO as the validation set, we built a consistency model based on 101 algorithm combinations and calculated the average C-index of each model across all cohorts to evaluate the predictive performance (Fig. 4B). Among the 101 models, the RSF algorithm exhibited the highest average C-index, and was thus used to construct the final model. The Cox boost algorithm identified the most valuable SPRGs, while the stepwise Cox algorithm filtered out the most significant models, resulting in the construction of CMLS, which included all 6 identified SPRGs. After calculating the CMLS scores for each sample in all cohorts, survival curves were drawn. Patients with high CMLS in both TCGA and meta-GEO cohorts showed poorer clinical outcomes (*P* < 0.0001and *P* = 1.1e-04, respectively) (Fig. 4D, E). These results were validated in the TCGA cohort and meta-GEO cohort through single-gene expression analysis and OS survival analysis (Additional file 2: Figure S9, S20).

### Multivariate cox analysis of CMLS

Through multivariate Cox analysis, we identified that CMLS consists of three risk factors (*CAP2*, *SELL*, *LAPTM5*) and three protective factors (*GZMA*, *FCER1G*, and *LYZ*) (Fig. 4C). Further investigation revealed that *CAP2* is strongly associated with malignant melanoma, with previous studies suggesting its potential as an independent diagnostic factor [32].

### Immune characteristics associated with CMLS

**Fig. 5:**
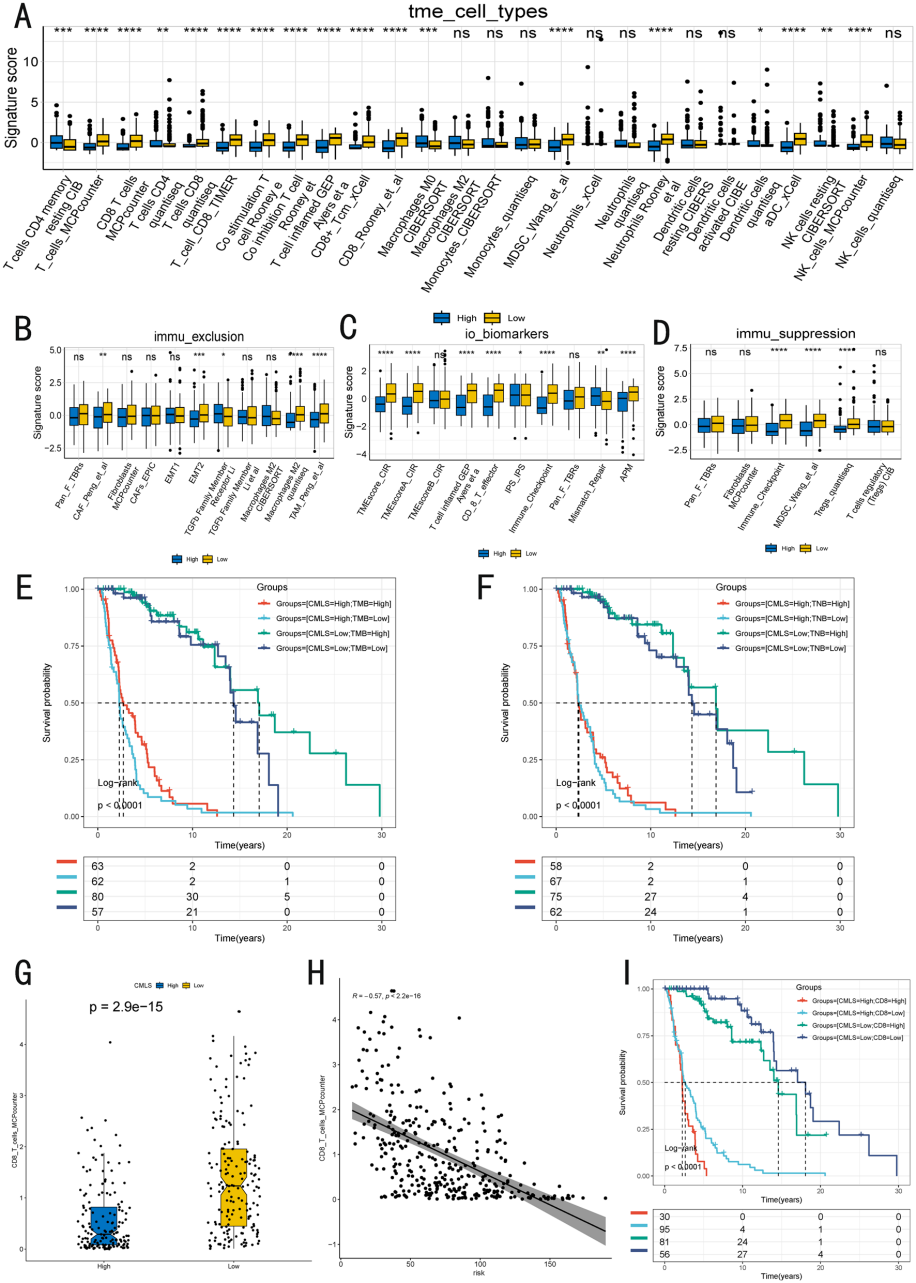
The TME-related molecular characteristics of high- and low-CMLS patients. **A** The distribution of TME immune cell type signatures between high-and low-CMLS patients. **B** The distribution of immune exclusion signatures between high- and low-CMLS patients. **C** The distribution of immunotherapy biomarkers between high and low-CMLS patients. **D** The distribution of immune suppression signatures between high-and low CMLS patients. **E**, **F** Survival analysis combined CMLS with TMB and TNB. **G** The distribution of CD8 + T cells between high- and low-CMLS patients. **H** The relationship between CMLS and CD8 + T cells. **I** Survival analysis combined CMLS with CD8 + T cells

We conducted a comprehensive analysis of the tumor microenvironment (TME) in SKCM (Fig. 5A). Compared to the CMLS high group, the CMLS low group showed significantly more active CD8 + T cells, CD4 + T cells, co-stimulating T cells, neutrophils, and dendritic cells. In contrast, the high CMLS group exhibited active M0 macrophages, indicating that the high CMLS group was classified as a "cold tumor" phenotype, while the low CMLS group was more inclined to be a "hot tumor" phenotype. In terms of tumor exclusion (Fig. 5B), the low CMLS group exhibited more cancer-associated fibroblasts and tumor-associated macrophages, further supporting this classification. Regarding immuno-oncology biomarkers (io-biomarkers), the TME score, T cell infiltration, and antigen presentation machinery (APM) were significantly activated in the low CMLS group, while mismatch repair was relatively activated in the high CMLS group (Fig. 5C). This suggests that the low CMLS group has a stronger anti-tumor immune response, while the high CMLS group exhibits higher tumor malignancy. In the immune suppression profile (Fig. 5D), we found that the low CMLS group had higher expression of regulatory T cells (Tregs) and myeloid-derived suppressor cells (MDSCs).

### Tumor mutational burden (TMB), tumor neoantigen burden (TNB) and their implications

TMB is currently recognized as a biomarker for evaluating patient responses to immunotherapy, and it has shown promising prognostic value in several cancers. We analyzed the differences in TMB levels between the two groups and found no significant difference in early stages between high and low TMB within the low CMLS group (Fig. 5E). However, clear differentiation emerged in later stages. In the high CMLS group, it was difficult to predict outcomes based on TMB levels. TNB refers to the number of neoantigens generated by mutations in the tumor. Neoantigens are tumor-specific antigens caused by mutations that can be recognized by the immune system. In the TNB combined with CMLS risk analysis, similar results to the above were obtained (Fig. 5F).

### Correlation between CD8 + T cells and prognosis

To further investigate the relationship between CD8 + T cell expression and patient prognosis, we conducted a correlation analysis and survival curve plotting (Fig. 5G–I). We found the low-risk CMLS group has significantly more CD8 + T cells than the high-risk CMLS group (*P* = 2.9e-15) and a significant negative correlation between the risk score and the number of CD8 + T cells (R = -0.57, *P* = 2.2e-16). While CD8 + T cells were significantly activated in the low CMLS group, excessive activation was found to be detrimental to long-term survival.

### Immunotherapy and potential drug discovery

Given that CMLS is closely associated with patient prognosis, we utilized the Cancer Therapeutics Response Portal (CTRP) and Profiling Relative Inhibition Simultaneously in Mixture (PRISM) to screen for potential therapeutic drugs.

**Fig. 6:**
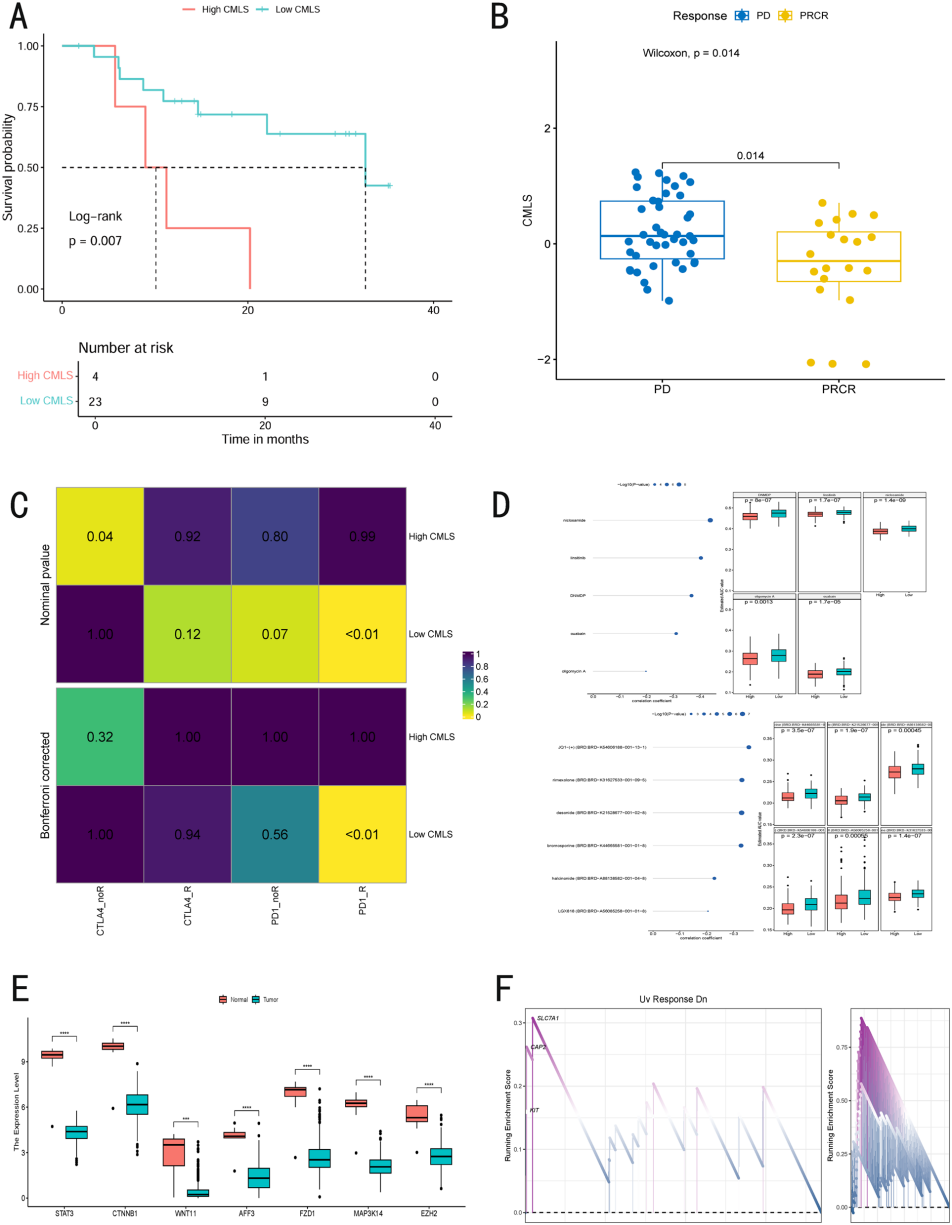
The value of CMLS in predicting immunotherapy response and potential drug discovery in MUC patients. **A** Survival analysis of high risk- and low risk-CMLS groups in patients receiving anti-PD-1 immunotherapy. **B** The distribution of CMLS in PD and PRCR immunotherapy response groups. **C** The subclass mapping algorithm predicts response to immunotherapy between high risk-and low risk-CMLS groups. **D** The correlation and differential analysis of drug sensitivity for potential drugs screened from the CTRP and PRISM datasets. **E** The unpaired differential expression analyses for potential target gene of screened drugs in normal and tumor tissue. *P < 0.05, **P < 0.01, ***P < 0.001. **F** Discovery of pathways significantly activated in the high risk-CMLS group through the GSEA algorithm

To enhance the robustness of our predictions, we included a cohort of melanoma patients treated with PD-1 inhibitors from the GSE91061 and classified them into high- and low-CMLS groups based on their CMLS expression levels (Fig. 6A). The results indicated that patients in the low-CMLS group exhibited better prognoses. Additionally, patients were classified into two groups based on treatment outcomes: progressive disease (PD) and partial/complete response. We observed that patients in the PD group exhibited significantly higher CMLS scores than those in the PRCR group, suggesting that high CMLS may be associated with resistance to PD-1 inhibitor therapy (*P* = 0.014) (Fig. 6B). Further analysis using the Subtype Mapping Algorithm confirmed that this group was significantly more responsive to programmed cell PD-1 inhibitors (Bonferroni-corrected* P* < 0.01) (Fig. 6C).

Based on previous studies, we systematically explored potential therapeutic strategies for high-CMLS patients (Fig. 6D). From the CTRP database, we identified three potential therapeutic compounds: Niclosamide, Linsitinib, DNMDP. Meanwhile, from the PRISM database, we identified four additional potential drugs: JQ1 (JQ1-(+), BRD: BRD-K54606188-001-13-1), Rimexolone (BRD: BRD-K31627533-001-09-5), Desonide (BRD: BRD-K21528677-001-02-8), Bromosporine (BRD: BRD-K44665581-001-01-8).

We incorporated normal samples from the GSE146517 dataset to validate the potential drug targets and merged them with the TCGA cohort. Using an unpaired t-test, we generated boxplots of gene expression (Fig. 6E), revealing that the expression of potential drug target genes was significantly higher in the normal group than in the cancer group, further supporting their therapeutic potential in melanoma.

Finally, we performed GSEA enrichment analysis on differentially expressed genes between high- and low-CMLS groups in the TCGA cohort (Fig. 6F). The results showed that CMLS key gene *CAP2* was significantly enriched in the "UV exposure downregulation pathway", suggesting that *CAP2* may play a specific role in UV-induced cellular responses. This finding provides new insights into the pathogenesis of melanoma and potential therapeutic strategies.

### Analysis of CMLS at the single-cell level

**Fig. 7:**
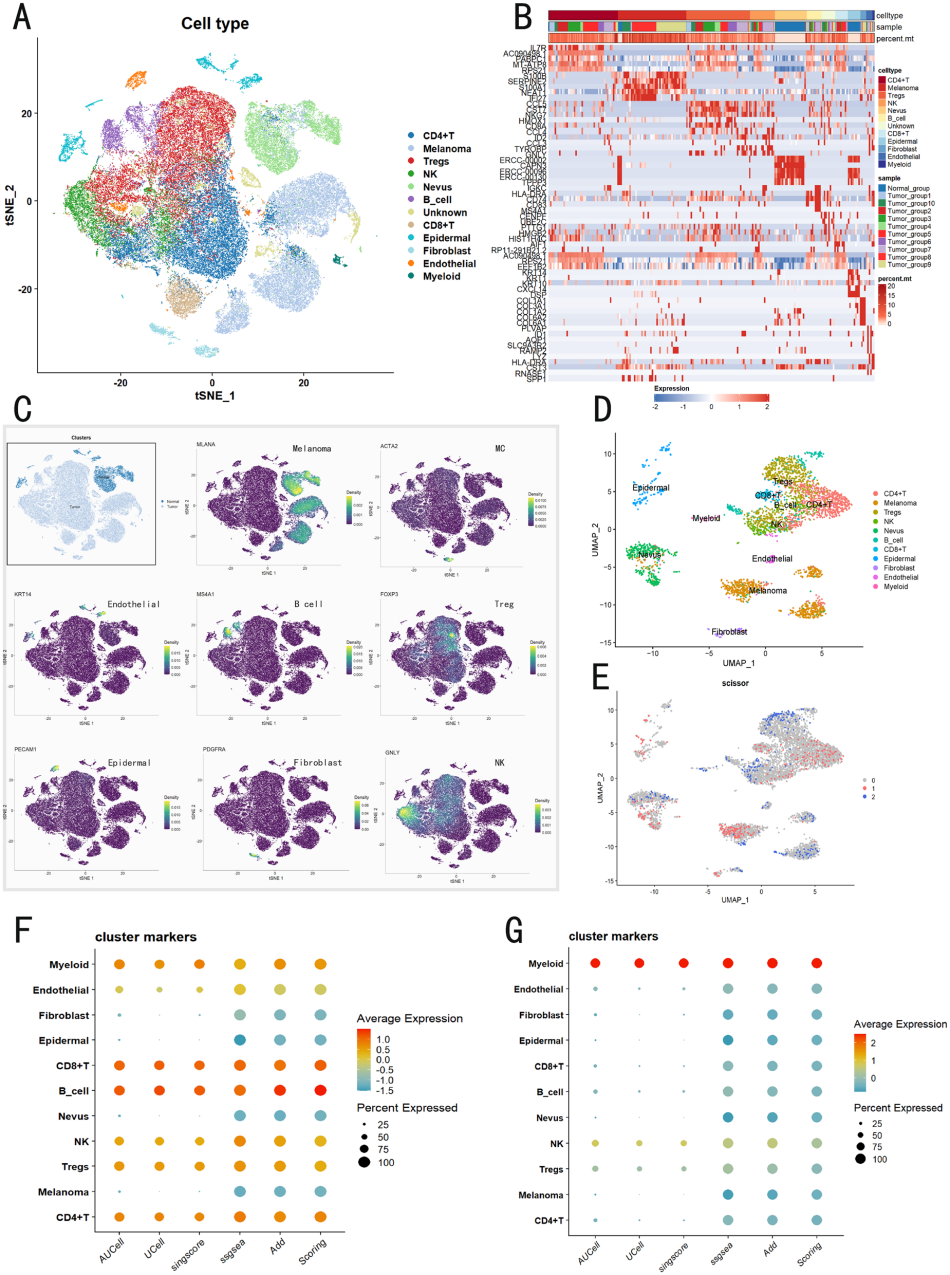
Single-cell analysis and CMLS scoring results.** A** TSNE plot demonstrating the cell distribution from four skin malignant melanoma samples, six lymphatic samples and 22 normal nevus samples, color-coded by the annotated cell types. **B** The top-5 genes of each cell type, along with their sample sources and mitochondrial gene expression levels. **C** Feature plots presenting classical marker genes for the annotated cell types. **D** UMAP plot demonstrating the cell distribution, color-coded by the annotated cell types. **E** UMAP visualization of the Scissor-selected cells across cell populations. **F** Evaluate the expression of risk factors in each cell type using five algorithms and their average scores. **G** Evaluate the expression of protective factors in each cell type using five algorithms and their average scores. A total of cells were classified into major immune, malignant, and stromal populations, including CD4⁺ T cells (n = 14,476), melanoma cells (n = 14,166), regulatory T cells (n = 13,247), NK cells (n = 5145), nevus cells (n = 6646), B cells (n = 3022), CD8⁺ T cells (n = 2589), epidermal cells (n = 2600), fibroblasts (n = 1233), endothelial cells (n = 1223), and myeloid cells (n = 441), with a small fraction of unclassified cells (n = 2903). All major cell populations were derived from multiple tumors, indicating that the identified clusters reflect biological heterogeneity rather than tumor-specific effects

We further investigated the expression patterns and underlying mechanisms of CMLS across different cellular subpopulations. Three datasets (GSE215120, GSE148190, and GSE151091) were collected, and skin malignant melanoma samples were selectively extracted from the GSE215120 dataset. The single-cell data were subsequently processed and annotated, resulting in an aggregated gene expression matrix comprising 67,691 cells and 50,436 genes. At a resolution of 0.5, 18 distinct cell clusters were identified and visualized using t-distributed Stochastic Neighbor Embedding (t-SNE) (Fig. 7A). To delineate the major cell types, each cluster was annotated using cluster-specific marker genes. Initially, malignant melanoma cells and normal nevus cells were distinguished based on the melanoma-specific marker MLANA and sample provenance. The remaining clusters were classified into nine non-tumor cell types, and we confirmed that the key cell types robustly express their well-established marker genes, demonstrating high specificity (Fig. 7B, C).

To eliminate the interaction effects between protective and risk genes, we divided CMLS into a protective group and a risk group, with each consisting of protective or risk genes, respectively. We then applied five single-cell scanning techniques to calculate the CMLS score, followed by averaging the scores of these five techniques, termed “scoring”. The results revealed that the risk group showed significant activation in B cells and CD8 + T cells (Fig. 7F), particularly the former. Additionally, there was some expression in other immune cells, but expression in non-immune cells was minimal. In contrast, the protective group showed significant expression only in myeloid cells (Fig. 7G).

**Fig. 8:**
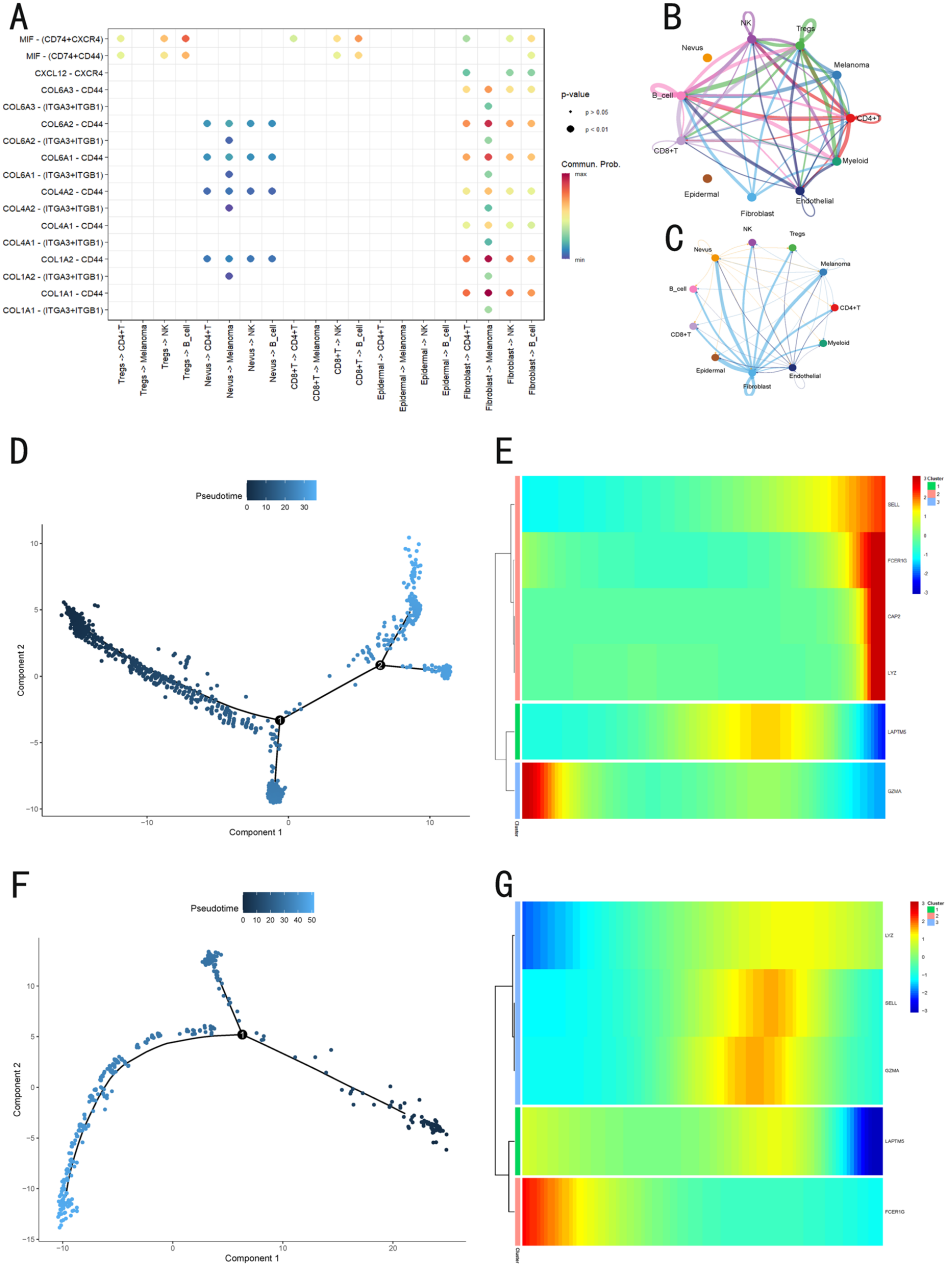
Cell-cell communication analysis, CMLS scoring and trajectory analysis results.** A** Cell-cell pathway activation status. **B**, **C** Cell-cell communication, **D** B cell pseudotime developmental trajectory, **E** Temporal expression of CMLS genes in B cells. **F** Myeloid cell pseudotime developmental trajectory. **G** Temporal expression of CMLS genes in myeloid cells

Next, we explored intercellular communication (Fig. 8A–C) and discovered that the Tregs cells activated the macrophage migration inhibitory factor (MIF) pathway with B cells, suggesting a potential immunosuppressive role for B cells. Furthermore, the COL1A2, COL1A1, and CD44 pathways were significantly activated between fibroblasts and melanoma cells. COL1A1 and COL1A2 pathways are major components of the extracellular matrix, and CD44 pathways is a critical cell adhesion molecule. Their activation suggests extracellular matrix remodeling, which enhances the migration and invasion abilities of melanoma cells, potentially correlating with poor prognosis.

Furthermore, trajectory inference analysis was performed on our scRNA-seq data using Monocle package to explore the dynamics of CMLS expression at single-cell resolution. Based on the CMLS scores derived from the five scanning techniques, B cells and myeloid cells were selected as target populations. In B cells (Fig. 8D, E), *GZMA* was significantly activated during early developmental stages, while *CAP2*, *FCER1G*, *LYZ*, and *SELL* were upregulated at later stages. In myeloid cells (Fig. 8F, G), *FCER1G* showed early activation, whereas *LAPTM5* exhibited pronounced downregulation in later stages, with *CAP2* remaining unexpressed throughout the developmental trajectory.

### Integrating bulk and single-cell sequencing data to identify phenotype-related subpopulations

To integrate the previous TCGA data with single-cell analysis, we used the Single-Cell Identification of Subpopulations with bulk Sample phenOtype coRrelation (Scissor) method for joint analysis. This algorithm automatically selects cell subpopulations from single-cell data that are most responsible for phenotype differences, using bulk data and phenotype information. Then, we evaluated the single-cell data using the Scissor package (Fig. 7D, E). We found that Scissor + cells were highly concentrated in melanoma cells, with some aggregation also observed in CD4 + T cells and normal nevi cells. This suggests a strong correlation between melanoma cells and high-risk CMLS.

### Spatial transcriptomic analysis of CMLS

**Fig. 9:**
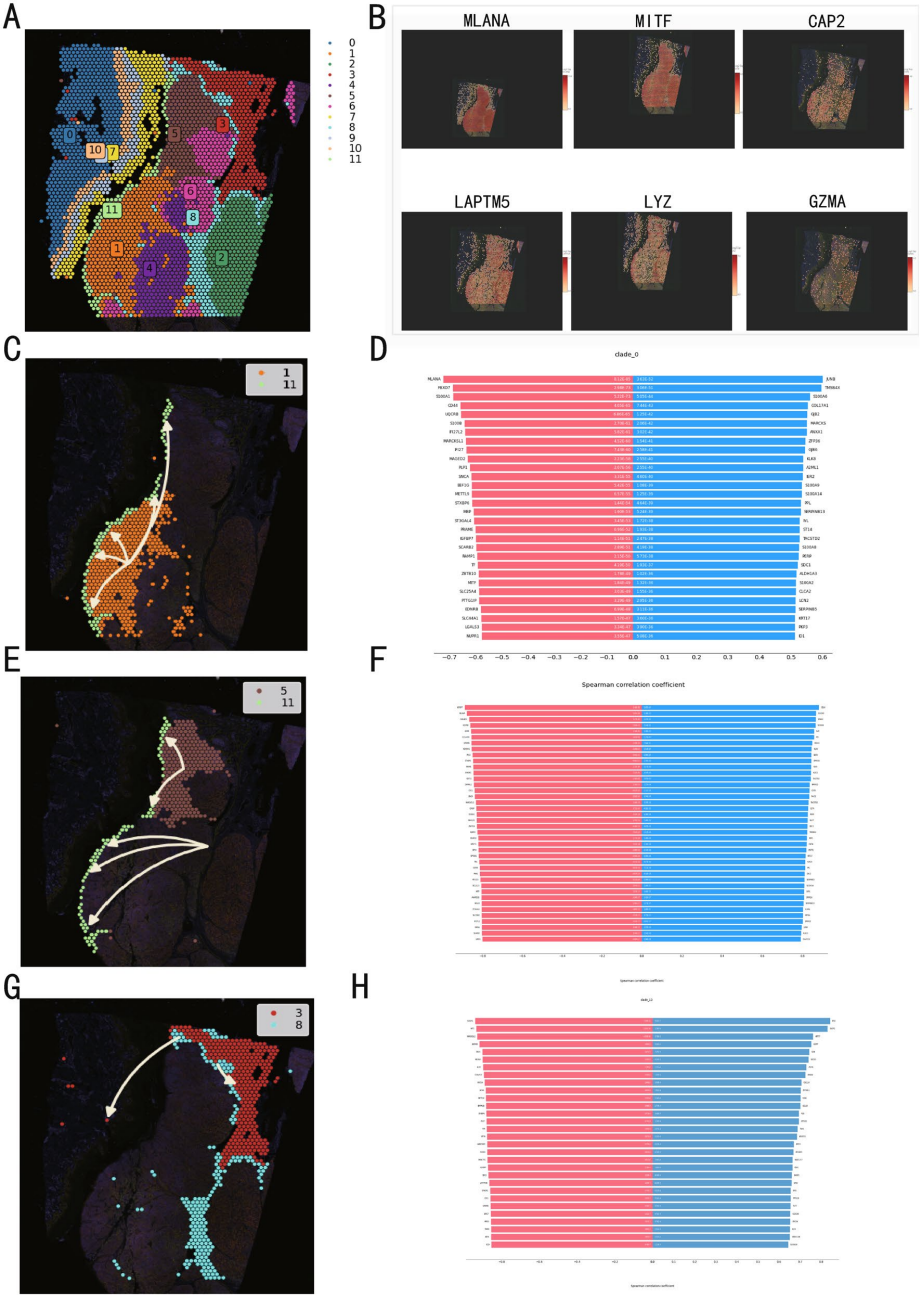
The ST analysis results.** A** The spatial images of primary malignant melanoma. **B** The spatial expression of CMLS genes. **C**, **D** The developmental trajectory from the first cluster to the eleventh cluster, along with the gene expression changes during this process. **E**, **F** The developmental trajectory from the fifth cluster to the eleventh cluster, along with the gene expression changes during this process. **G**, **H** The developmental trajectory from the eighth cluster to the third cluster, along with the gene expression changes during this process

To investigate the spatial expression of CMLS, we collected spatial transcriptomic data from primary malignant melanoma (Human Melanoma, IF Stained (FFPE)) and brain metastatic melanoma (GSM5420750). We examined the expression patterns of CMLS genes and characteristic melanoma genes in primary malignant melanoma using Loupe Browser (Fig. 9B). *MLANA* and *MITF* exhibited significant expression in the tumor nest, with rapid decrease in expression at the tumor margin. In contrast, *CAP2* and *LAPTM5* showed increased expression at the tumor margin, suggesting that these genes are upregulated in newly formed tumor cells and may contribute to their invasiveness. Interestingly, *LYZ* also displayed a prominent peripheral expression pattern, possibly linked to immune cell infiltration at the tumor periphery. *GZMA* showed low expression in the tumor nest.

**Fig. 10:**
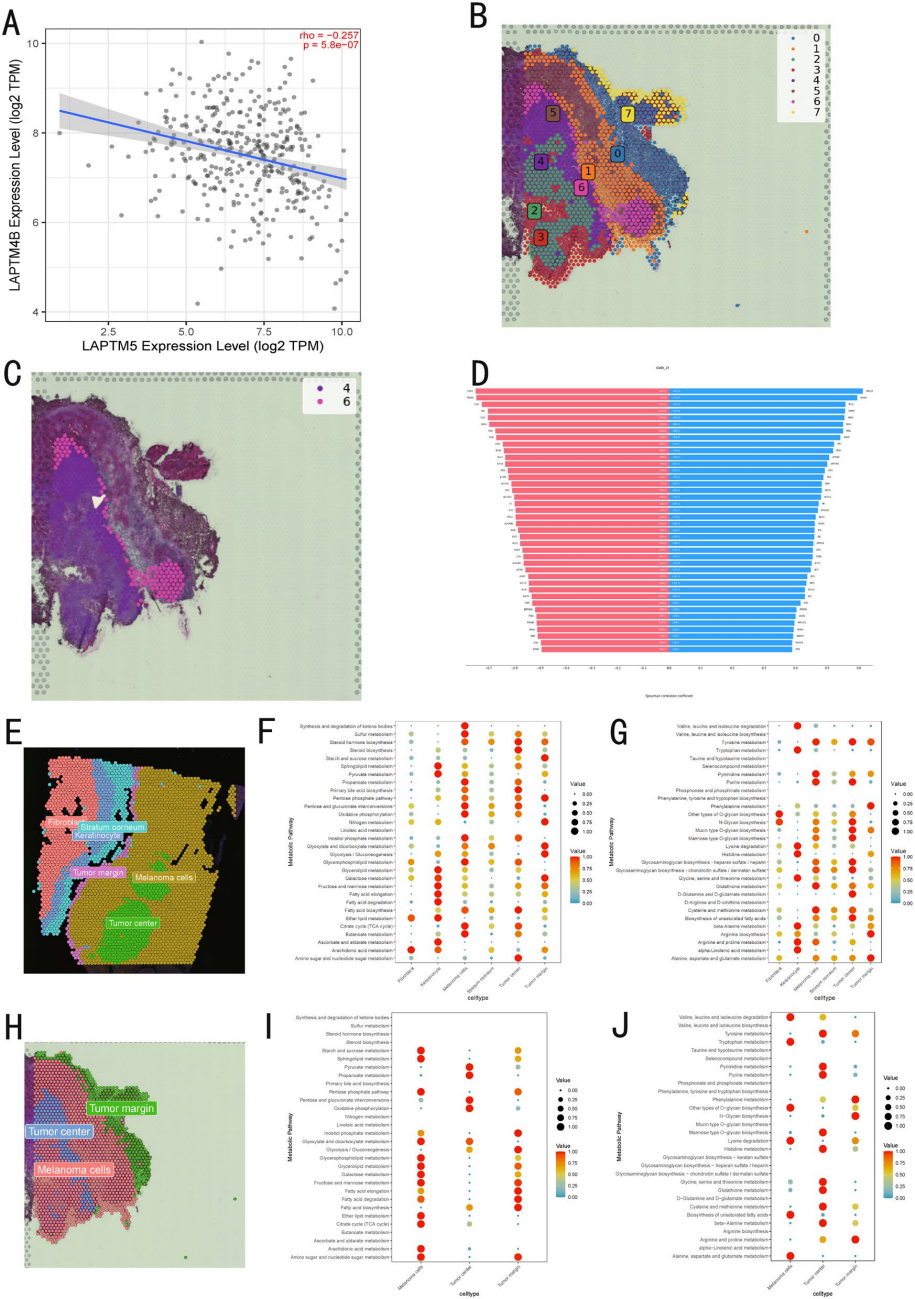
The additional ST analysis results. **A** The relationship between *LAPTM5* and* LAPTM4B*. **B** The spatial images of metastasis in malignant melanoma. **C**, **D** The developmental trajectory from the fourth cluster to the sixth cluster, along with the gene expression changes during this process. **E** Cell annotation of primary malignant melanoma. **F** Cell annotation of metastatic malignant melanoma. **G**, **H** Related metabolism of primary malignant melanoma. **I**, **J** Related metabolism of metastatic malignant melanoma

We estimated intercellular interactions using tool stLearn [33], treating the primary tumor nest as the developmental origin (cluster 4) (Fig. 9A). Spatial pseudotime trajectory analysis was then performed using tool stLearn. We observed that in the development from cluster 1 to cluster 11 (Fig. 9C) and from cluster 5 to cluster 11 (Fig. 9E), *MLANA* and *MITF* showed a significant negative correlation (Fig. 9D, F). Cluster 3 appeared not only in the deep tumor area but also sporadically in normal tissue (Fig. 9G), suggesting it could represent metastatic melanoma cells. During the development from cluster 8 to cluster 3, we found that besides *MLANA*, *LAPTM4B* also showed a significant decrease (Fig. 9H). We further validated this by observing the negative correlation between *LAPTM5* and *LAPTM4B* in TIMER2.0 (http://timer.cistrome.org) (Fig. 10A), which confirmed that LAPTM5 expression is elevated in primary newly formed tumor cells.

Subsequently, we used the sixth cluster of the secondary tumor nest as the developmental origin (Fig. 10B) and found that during the outward tumor spread *MLANA* expression was enhanced, while the macrophage migration inhibitory factor (MIF) pathway was significantly activated (Fig. 10C, D). However, *LAPTM5* showed a negative correlation, indicating that gene expression differs significantly between primary and secondary malignant melanoma during tumor development.

Moreover, at the histological level, both primary and metastatic melanoma tumor nests were subdivided into three regions—Tumor Center, Melanoma Cells, and Tumor Margin—for metabolic pathway analysis (Fig. 10E, F). In primary malignant melanoma (Fig. 10G, H), tumor cells generally exhibit strong tyrosine metabolism and pentose phosphate pathway activity, with glycolysis intensifying gradually from the center to the margin. Conversely, N-glycan biosynthesis and mannose-type O-glycan biosynthesis decrease from the center to the margin, implying a reduction in cell adhesion capabilities. In metastatic melanoma (Fig. 10I, J), however, tyrosine metabolism is attenuated, while amino sugar and nucleotide sugar metabolism and fatty acid elongation pathways progressively intensify during tumor development. These changes suggest alterations in cell surface glycosylation and the production of more complex lipid molecules via fatty acid chain elongation.

## Discussion

Our study identified two distinct molecular subtypes of SKCM using multimodal data integration with the MOVICS package: CS1 and CS2. CS2 was enriched in immune-suppressive pathways like WNT-β signaling with a better prognosis, while CS1 exhibited higher activation of the PI3K pathway, DNA repair mechanisms, and downregulated fibrosis, indicating greater tumor invasiveness. TWAS analysis results combined the findings from TCGA-SKCM and the meta-cohort, identifying six significant prognostic-related genes (SPRGs) (*CAP2*, *SELL*, *LAPTM5*, *GZMA*, *FCER1G*, and *LYZ*). The CMLS prognostic model, based on six SPRGs, stratified patients into high-risk (poorer survival) and low-risk groups. Single-cell and spatial transcriptomic analyses further validated CMLS results, revealing differences in gene expression, tumor microenvironment interactions, and progression trajectories, providing insights into the molecular mechanisms driving SKCM.

*CAP2*, *LAPTM5*, *LYZ*, and *FCER1G* were already reported in previous SKCM studies. Adenylate cyclase-associated protein (CAP) is a well-conserved actin-monomer-binding protein, initially identified in yeast as a factor involved in RAS-activated adenylate cyclase activity [34].Notably, *CAP2* overexpression has been proposed as a novel prognostic marker in melanoma, with studies showing that its expression increases progressively during tumor progression [32, 35]. Lysosomal-associated transmembrane protein 5 (LAPTM5) is a molecular partner of CD1e, a protein involved in lipid antigen presentation by dendritic cells [36]. Initially, *LAPTM5* expression was believed to be limited to the hematopoietic lineage and embryonic stem cells [37]. However, subsequent studies have revealed its upregulation in glial cells in response to neuronal apoptosis [38]. Additionally, *LAPTM5* has been found to be expressed in benign neuroblastomas [39], and it is significantly upregulated in two melanoma cell lines [36]. These findings suggest that *LAPTM5* may play a role in tumor biology, particularly in melanoma. Our study supports these findings, as we also observed significant upregulation of *LAPTM5* in melanoma, further implicating it as a potential biomarker for cancer progression. *LYZ* encodes a protein with antimicrobial activity against bacterial peptidoglycan, playing a role in innate immunity. In SKCM, increased *LYZ* expression has been associated with macrophage infiltration [40]. Recently, it has been used to isolate synthetic bacterial vesicles, which, when combined with melanoma extracellular vesicles, contribute to tumor regression in melanoma-bearing mice [41]. Its prognostic value has also been confirmed in previous datasets [42], suggesting its potential as a biomarker for patient outcomes. Similarly, *LYZ* significantly downregulated in melanoma in our study. *FCER1G*, located on chromosome 1q23.3 [43], encodes a key component of the high-affinity immunoglobulin E (IgE) receptor and the interleukin-3 receptor complex. Studies suggest its tumor-suppressive role in multiple myeloma [44] and a negative association with melanoma [45, 46]. Consistently, our study found *FCER1G* to be significantly downregulated in melanoma, indicating its potential role in reducing melanoma risk and serving as a biomarker for cancer progression.

Interestingly, *SELL* and *GZMA* were not reported in previous SKCM studies. L-selectin (SELL) is a type of leukocyte adhesion molecule involved in the interaction between leukocytes and vascular endothelium [47]. It regulates immune cell trafficking and is upregulated in various cancers [48], making it a potential immunotherapy target. In a mouse model, *SELL* overexpression in T cells enhanced tumor infiltration and proliferation, improving the chance of tumor control [49]. Previous experiments constructed an aneuploidy related riskscore (ARS) model that included *SELL*, and patients in the low ARS group were more sensitive to immunotherapy [50]. Similarly, our study found *SELL* significantly upregulated in melanoma, reinforcing its role as a biomarker for cancer progression. Another gene, Granzyme A (GZMA), secreted by natural killer (NK) cells, has drawn significant attention as a biomarker for assessing cancer immunotherapy efficacy [51]. It has been identified as a potential prognostic marker across various cancer types [52, 53]. Previous studies have highlighted its significance in breast cancer and lung squamous cell carcinoma, where higher *GZMA* expression is associated with improved survival outcomes [54, 55]. However, evidences suggest a pro-proliferative role of extracellular *GZMA* in colorectal cancer progression [56, 57]. In our study, we observed *GZMA* downregulation in melanoma. Further investigations are warranted to elucidate the context-dependent role of *GZMA* across different cancer types. Future studies should explore the underlying regulatory mechanisms driving its differential expression, assess its impact on tumor microenvironment modulation in melanoma, and determine whether its functional role is influenced by specific immune or stromal interactions. Experimental approaches such as single-cell RNA sequencing, spatial transcriptomics, and functional assays in melanoma models will be essential to clarify these discrepancies and uncover potential therapeutic implications.

Additionally, our study provides important insights into the biological significance of SKCM subtypes. CS1 exhibits higher activation of PI3K pathways, DNA repair mechanisms, and downregulation of fibrosis, leading to more aggressive tumors, while CS2 is associated with better survival outcomes, exhibiting abundant immunosuppressive pathways such as WNT-β signaling. These findings suggest that the different molecular characteristics of CS1 and CS2 lead to their different prognostic implications. Furthermore, the immune microenvironment of CS2 is characterized by immune checkpoint activation and elevated immune cell infiltration, which may enhance its responsiveness to immunotherapy. These observations are consistent with previous studies emphasizing the prognostic impact of immune activity on SKCM. Our findings also suggest that the CMLS model based on the six SPRGs is an effective prognostic tool for stratifying patients into high- and low-risk groups. This stratification has potential clinical utility in guiding treatment decisions and improving patient management. Notably, we found that the immunosuppressive status of the good prognosis group was significantly higher than that of the poor prognosis group. This phenomenon may be related to the high activation of the immune system in the former group, in which the organism maintains immune homeostasis through a negative feedback regulatory mechanism during a strong immune response, leading to enhanced immunosuppression. This result suggests that the good prognosis group may have experienced stronger immune activation, but it was also accompanied by a balanced process of immune regulation.

A primary advantage of our study is the comprehensive integration of multi-omics data, including mRNA, lncRNA, miRNA, genomic mutation, and methylation profiles, which ensures robust classification of SKCM subtypes. The consistency of our subtypes across multiple independent datasets and clustering algorithms further demonstrates the reliability of our findings. In addition, the application of single-cell and spatial transcriptome analyses provided additional levels of validation, allowing us to gain insight into tumor heterogeneity and microenvironmental interactions.

Nonetheless, we must also recognize several limitations. First, this study is based on retrospective public datasets, which may introduce unavoidable selection bias, and prospective validation in independent clinical cohorts will be required to confirm generalizability. Second, although the molecular subtypes and the CMLS signature were derived using established workflows and validated across multiple datasets and analytical layers, they were identified through computational integration rather than experimental perturbation. Besides CS1/CS2 and CMLS show partial overlap with previously reported melanoma molecular subtypes and prognostic signatures, this consistency across independent methods and datasets strengthens the robustness and reproducibility of these biological patterns rather than diminishing their significance or claiming entirely novel mechanisms. Third, the single-cell and spatial transcriptomic analyses were used to provide biological context and cross-scale support for bulk-level findings, particularly in relation to tumor–immune interactions and metabolic heterogeneity. However, the limited number of samples, heterogeneous tissue origins, and lack of dynamic modeling restrict their ability to support definitive mechanistic or causal conclusions. In addition, we evaluated the clinical relevance of CMLS in the context of immunotherapy. Validation in the GSE91061 cohort supports the predictive value of CMLS for PD-1 blockade response, particularly in identifying non-responders. However, we acknowledge that the sample size of this cohort is relatively modest. While these trends are biologically consistent, the primary objective of CMLS remains prognostic stratification rather than treatment-specific response prediction; therefore, these findings warrant further verification in larger, prospective multi-center immunotherapy cohorts. Finally, functional perturbation experiments of key SPRGs, dynamic systems modeling of cell–cell communication networks, and benchmarking against all previously published melanoma signatures were beyond the scope of the present study, which emphasizes multi-omics consensus integration, cross-cohort validation, and interpretability. These extensions warrant future dedicated investigation.

Although CS1/CS2 and CMLS show partial overlap with previously reported melanoma molecular subtypes and prognostic signatures, this consistency across independent methods and datasets strengthens the robustness and reproducibility of these biological patterns rather than diminishing their significance. Our study focuses on multi-omics integration and cross-cohort validation; therefore, functional perturbation experiments and dynamic systems modeling were beyond the current scope and warrant future investigation. Additionally, while advanced machine learning or deep learning approaches may provide complementary validation, our consensus-based framework emphasizes interpretability and robustness across platforms.

Finally, although key pathways associated with prognosis and immune phenotypes were identified, functional experiments and real-world clinical validation are still required to establish causality and assess the utility of CMLS in guiding treatment strategies.Future studies should focus on validating in preclinical models the specific roles of the key biomarkers we identified, such as *CAP2*, *GZMA*, and *SELL*, in SKCM progression. In addition, further exploration of the tumor microenvironment at single-cell resolution will contribute to a deeper understanding of the cell–cell interactions that drive SKCM heterogeneity. Prospective cohort studies are also needed to confirm the value of CM prognostic models in the clinic and their potential in personalized therapy. In addition, a promising research direction is the development of targeted therapies against identified pathways. Considering the differences in PI3K and WNT-β pathway activation between CS1 and CS2, therapeutic strategies could be tailored to the specific vulnerabilities of the different subtypes. Meanwhile, exploring the effects of combination therapies such as immune checkpoint inhibitors and PI3K pathway inhibitors may provide new treatment avenues for SKCM patients.

## Conclusion

Our findings have important clinical implications for the treatment of SKCM. The identification of different molecular subtypes lays the foundation for personalized therapeutic strategies. patients with CS2 which targets WNT-β pathway activation have a favorable immune profile and may benefit more from immune checkpoint blockade therapies, whereas patients with CS1 may require alternative therapeutic approaches targeting PI3K signaling or fibrosis-related mechanisms. In addition, the CMLS model based on six prognostic genes, *CAP2*, *SELL*, *LAPTM5*, *GZMA*, *FCER1G*, and *LYZ*, provides a promising tool for risk stratification in SKCM and may serve as a therapeutic target. Future studies should explore the incorporation of these findings into a personalized medicine framework to further enhance SKCM management.

## Supplementary Information

Supplementary Material 1.

Supplementary Material 2.

## Data Availability

Data used in this study are available from the previous studies and listed in Additional file 1: Table S1.

## Code availability

Multi-omics clustering: MOVICS (v0.99.17; https://github.com/xlucpu/MOVICS), Single-cell analysis: Seurat (v4.3.0.1; https://github.com/satijalab/seurat), CellChat (v1.1.3; https://github.com/jinworks/CellChat), Monocle (v2.30.1; https://github.com/cole-trapnell-lab/monocle-release), Spatial transcriptomics: stLearn (https://github.com/BiomedicalMachineLearning/stLearn), Metabolic analysis: scMetabolism (v0.2.1; https://github.com/wu-yc/scMetabolism), TWAS analysis: PrediXcan (https://github.com/hakyimlab/PrediXcan), Essential scripts for implementing machine learning-based integrative procedure in multiple independent datasets are available on the Github website (https://github.com/Zaoqu-Liu/IRLS).

## References

[1] Long GV, Swetter SM, Menzies AM, Gershenwald JE, Scolyer RA. Cutaneous melanoma. Lancet. 2023;402(10400):485–502. 37499671 10.1016/S0140-6736(23)00821-8

[2] Elder DE, Bastian BC, Cree IA, Massi D, Scolyer RA. The 2018 world health organization classification of cutaneous, mucosal, and uveal melanoma: detailed analysis of 9 distinct subtypes defined by their evolutionary pathway. Arch Pathol Lab Med. 2020;144(4):500–22. 32057276 10.5858/arpa.2019-0561-RA

[3] Whiteman DC, Green AC, Olsen CM. The growing burden of invasive melanoma: projections of incidence rates and numbers of new cases in six susceptible populations through 2031. J Invest Dermatol. 2016;136(6):1161–71. 26902923 10.1016/j.jid.2016.01.035

[4] Robinson M, Primiero C, Guitera P, Hong A, Scolyer RA, Stretch JR, et al. Evidence-based clinical practice guidelines for the management of patients with Lentigo Maligna. Dermatology (Basel, Switzerland). 2020;236(2):111–6. 31639788 10.1159/000502470

[5] Eggermont AM, Spatz A, Robert C. Cutaneous melanoma. Lancet. 2014;383(9919):816–27. 24054424 10.1016/S0140-6736(13)60802-8

[6] Spieth K, Kovács A, Wolter M, Bug R, Kaufmann R, Gille J. Topical imiquimod: effectiveness in intraepithelial melanoma of oral mucosa. Lancet Oncol. 2006;7(12):1036–7. 17138226 10.1016/S1470-2045(06)70979-2

[7] Schadendorf D, van Akkooi ACJ, Berking C, Griewank KG, Gutzmer R, Hauschild A, et al. Melanoma. Lancet (London, England). 2018;392(10151):971–84. 30238891 10.1016/S0140-6736(18)31559-9

[8] Klobuch S, Seijkens TTP, Schumacher TN, Haanen J. Tumour-infiltrating lymphocyte therapy for patients with advanced-stage melanoma. Nat Rev Clin Oncol. 2024;21(3):173–84. 38191921 10.1038/s41571-023-00848-w

[9] Luke JJ, Flaherty KT, Ribas A, Long GV. Targeted agents and immunotherapies: optimizing outcomes in melanoma. Nat Rev Clin Oncol. 2017;14(8):463–82. 28374786 10.1038/nrclinonc.2017.43

[10] Carlino MS, Larkin J, Long GV. Immune checkpoint inhibitors in melanoma. Lancet (London, England). 2021;398(10304):1002–14. 34509219 10.1016/S0140-6736(21)01206-X

[11] Leonardi GC, Candido S, Falzone L, Spandidos DA, Libra M. Cutaneous melanoma and the immunotherapy revolution (Review). Int J Oncol. 2020;57(3):609–18. 32582963 10.3892/ijo.2020.5088 PMC7384846

[12] Zaretsky JM, Garcia-Diaz A, Shin DS, Escuin-Ordinas H, Hugo W, Hu-Lieskovan S, et al. Mutations associated with acquired resistance to PD-1 blockade in melanoma. N Engl J Med. 2016;375(9):819–29. 27433843 10.1056/NEJMoa1604958 PMC5007206

[13] Hossain SM, Carpenter C, Eccles MR. Genomic and epigenomic biomarkers of immune checkpoint immunotherapy response in melanoma: current and future perspectives. Int J Mol Sci. 2024. 10.3390/ijms25137252. 39000359 10.3390/ijms25137252 PMC11241335

[14] Colaprico A, Silva TC, Olsen C, Garofano L, Cava C, Garolini D, et al. TCGAbiolinks: an R/Bioconductor package for integrative analysis of TCGA data. Nucleic Acids Res. 2016;44(8):e71. 26704973 10.1093/nar/gkv1507 PMC4856967

[15] Haunsberger SJ, Connolly NM, Prehn JH. miRNAmeConverter: an R/bioconductor package for translating mature miRNA names to different miRBase versions. Bioinformatics. 2017;33(4):592–3. 27797767 10.1093/bioinformatics/btw660

[16] Lu X, Meng J, Zhou Y, Jiang L, Yan F. MOVICS: an R package for multi-omics integration and visualization in cancer subtyping. Bioinformatics. 2021;36(22–23):5539–41. 33315104 10.1093/bioinformatics/btaa1018

[17] Hänzelmann S, Castelo R, Guinney J. GSVA: gene set variation analysis for microarray and RNA-seq data. BMC Bioinformatics. 2013;14:7. 23323831 10.1186/1471-2105-14-7 PMC3618321

[18] Lu X, Meng J, Su L, Jiang L, Wang H, Zhu J, et al. Multi-omics consensus ensemble refines the classification of muscle-invasive bladder cancer with stratified prognosis, tumour microenvironment and distinct sensitivity to frontline therapies. Clin Transl Med. 2021;11(12):e601. 34936229 10.1002/ctm2.601 PMC8693439

[19] Gamazon ER, Wheeler HE, Shah KP, Mozaffari SV, Aquino-Michaels K, Carroll RJ, et al. A gene-based association method for mapping traits using reference transcriptome data. Nat Genet. 2015;47(9):1091–8. 26258848 10.1038/ng.3367 PMC4552594

[20] Bycroft C, Freeman C, Petkova D, Band G, Elliott LT, Sharp K, et al. The UK Biobank resource with deep phenotyping and genomic data. Nature. 2018;562(7726):203–9. 30305743 10.1038/s41586-018-0579-z PMC6786975

[21] Liu Z, Liu L, Weng S, Guo C, Dang Q, Xu H, et al. Machine learning-based integration develops an immune-derived lncRNA signature for improving outcomes in colorectal cancer. Nat Commun. 2022;13(1):816. 35145098 10.1038/s41467-022-28421-6 PMC8831564

[22] Zeng D, Ye Z, Shen R, Yu G, Wu J, Xiong Y, et al. IOBR: multi-omics immuno-oncology biological research to decode tumor microenvironment and signatures. Front Immunol. 2021;12:687975. 34276676 10.3389/fimmu.2021.687975 PMC8283787

[23] Hoshida Y, Brunet JP, Tamayo P, Golub TR, Mesirov JP. Subclass mapping: identifying common subtypes in independent disease data sets. PLoS ONE. 2007;2(11):e1195. 18030330 10.1371/journal.pone.0001195 PMC2065909

[24] Trapnell C, Cacchiarelli D, Grimsby J, Pokharel P, Li S, Morse M, et al. The dynamics and regulators of cell fate decisions are revealed by pseudotemporal ordering of single cells. Nat Biotechnol. 2014;32(4):381–6. 24658644 10.1038/nbt.2859 PMC4122333

[25] Jin S, Guerrero-Juarez CF, Zhang L, Chang I, Ramos R, Kuan CH, et al. Inference and analysis of cell-cell communication using Cell Chat. Nat Commun. 2021;12(1):1088. 33597522 10.1038/s41467-021-21246-9 PMC7889871

[26] Sun D, Guan X, Moran AE, Wu LY, Qian DZ, Schedin P, et al. Identifying phenotype-associated subpopulations by integrating bulk and single-cell sequencing data. Nat Biotechnol. 2022;40(4):527–38. 34764492 10.1038/s41587-021-01091-3 PMC9010342

[27] Wu Y, Yang S, Ma J, Chen Z, Song G, Rao D, et al. Spatiotemporal immune landscape of colorectal cancer liver metastasis at single-cell level. Cancer Discov. 2022;12(1):134–53. 34417225 10.1158/2159-8290.CD-21-0316

[28] Zeng D, Li M, Zhou R, Zhang J, Sun H, Shi M, et al. Tumor microenvironment characterization in gastric cancer identifies prognostic and immunotherapeutically relevant gene signatures. Cancer Immunol Res. 2019;7(5):737–50. 30842092 10.1158/2326-6066.CIR-18-0436

[29] Zhang B, Wu Q, Li B, Wang D, Wang L, Zhou YL. M(6)A regulator-mediated methylation modification patterns and tumor microenvironment infiltration characterization in gastric cancer. Mol Cancer. 2020;19(1):53. 32164750 10.1186/s12943-020-01170-0 PMC7066851

[30] Chong W, Shang L, Liu J, Fang Z, Du F, Wu H, et al. M(6)A regulator-based methylation modification patterns characterized by distinct tumor microenvironment immune profiles in colon cancer. Theranostics. 2021;11(5):2201–17. 33500720 10.7150/thno.52717 PMC7797678

[31] Ritchie ME, Phipson B, Wu D, Hu Y, Law CW, Shi W, et al. Limma powers differential expression analyses for RNA-sequencing and microarray studies. Nucleic Acids Res. 2015;43(7):e47. 25605792 10.1093/nar/gkv007 PMC4402510

[32] Masugi Y, Tanese K, Emoto K, Yamazaki K, Effendi K, Funakoshi T, et al. Overexpression of adenylate cyclase-associated protein 2 is a novel prognostic marker in malignant melanoma. Pathol Int. 2015;65(12):627–34. 26374196 10.1111/pin.12351

[33] Pham D, Tan X, Balderson B, Xu J, Grice LF, Yoon S, et al. Robust mapping of spatiotemporal trajectories and cell-cell interactions in healthy and diseased tissues. Nat Commun. 2023;14(1):7739. 38007580 10.1038/s41467-023-43120-6 PMC10676408

[34] Ono S. The role of cyclase-associated protein in regulating actin filament dynamics - more than a monomer-sequestration factor. J Cell Sci. 2013;126(Pt 15):3249–58. 23908377 10.1242/jcs.128231 PMC3730240

[35] Li L, Fu LQ, Wang HJ, Wang YY. CAP2 is a valuable biomarker for diagnosis and prognostic in patients with gastric cancer. Pathol Oncol Res. 2020;26(1):273–9. 30047046 10.1007/s12253-018-0450-4

[36] Angénieux C, Waharte F, Gidon A, Signorino-Gelo F, Wurtz V, Hojeij R, et al. Lysosomal-associated transmembrane protein 5 (LAPTM5) is a molecular partner of CD1e. PLoS ONE. 2012;7(8):e42634. 22880058 10.1371/journal.pone.0042634 PMC3411835

[37] Adra CN, Zhu S, Ko JL, Guillemot JC, Cuervo AM, Kobayashi H, et al. LAPTM5: a novel lysosomal-associated multispanning membrane protein preferentially expressed in hematopoietic cells. Genomics. 1996;35(2):328–37. 8661146 10.1006/geno.1996.0364

[38] Origasa M, Tanaka S, Suzuki K, Tone S, Lim B, Koike T. Activation of a novel microglial gene encoding a lysosomal membrane protein in response to neuronal apoptosis. Brain Res Mol Brain Res. 2001;88(1–2):1–13. 11295227 10.1016/s0169-328x(01)00005-5

[39] Inoue J, Misawa A, Tanaka Y, Ichinose S, Sugino Y, Hosoi H, et al. Lysosomal-associated protein multispanning transmembrane 5 gene (LAPTM5) is associated with spontaneous regression of neuroblastomas. PLoS ONE. 2009;4(9):e7099. 19787053 10.1371/journal.pone.0007099 PMC2746316

[40] Currie GA. Serum lysozyme as a marker of host resistance. II. Patients with malignant melanoma, hypernephroma or breast carcinoma. Br J Cancer. 1976;33(6):593–9. 938609 10.1038/bjc.1976.96 PMC2025103

[41] Park KS, Svennerholm K, Crescitelli R, Lässer C, Gribonika I, Lötvall J. Synthetic bacterial vesicles combined with tumour extracellular vesicles as cancer immunotherapy. J Extracell Vesicles. 2021;10(9):e12120. 34262675 10.1002/jev2.12120 PMC8254025

[42] Strbenac D, Wang K, Wang X, Dong J, Mann GJ, Mueller S, et al. Melanoma explorer: a web application to allow easy reanalysis of publicly available and clinically annotated melanoma omics data sets. Melanoma Res. 2019;29(3):342–4. 31026248 10.1097/CMR.0000000000000533

[43] Küster H, Thompson H, Kinet JP. Characterization and expression of the gene for the human Fc receptor gamma subunit. Definition of a new gene family. J Biol Chem. 1990;265(11):6448–52. 2138619

[44] Fu L, Cheng Z, Dong F, Quan L, Cui L, Liu Y, et al. Enhanced expression of FCER1G predicts positive prognosis in multiple myeloma. J Cancer. 2020;11(5):1182–94. 31956364 10.7150/jca.37313 PMC6959079

[45] Liu J, Zhang X, Ye T, Dong Y, Zhang W, Wu F, et al. Prognostic modeling of patients with metastatic melanoma based on tumor immune microenvironment characteristics. Math Biosci Eng. 2022;19(2):1448–70. 35135212 10.3934/mbe.2022067

[46] Su S, Chhabra G, Ndiaye MA, Singh CK, Ye T, Huang W, et al. PLK1 and NOTCH positively correlate in melanoma and their combined inhibition results in synergistic modulations of key melanoma pathways. Mol Cancer Ther. 2021;20(1):161–72. 33177155 10.1158/1535-7163.MCT-20-0654 PMC7790869

[47] Sluiter W, Pietersma A, Lamers JM, Koster JF. Leukocyte adhesion molecules on the vascular endothelium: their role in the pathogenesis of cardiovascular disease and the mechanisms underlying their expression. J Cardiovasc Pharmacol. 1993;22(Suppl 4):S37-44. 7523771

[48] Choudhary D, Hegde P, Voznesensky O, Choudhary S, Kopsiaftis S, Claffey KP, et al. Increased expression of L-selectin (CD62L) in high-grade urothelial carcinoma: A potential marker for metastatic disease. Urol Oncol. 2015;33(9):387.e317-327. 10.1016/j.urolonc.2014.12.009 PMC4510033 25618296

[49] Watson HA, Durairaj RRP, Ohme J, Alatsatianos M, Almutairi H, Mohammed RN, et al. L-selectin enhanced T cells improve the efficacy of cancer immunotherapy. Front Immunol. 2019;10:1321. 31249570 10.3389/fimmu.2019.01321 PMC6582763

[50] Liu Y, Yuan Y, Chen T, Xiao H, Zhang X, Zhang F. Identification of aneuploidy-related gene signature to predict survival in head and neck squamous cell carcinomas. Aging Albany NY. 2023;15(22):13100–17. 37988195 10.18632/aging.205221 PMC10713391

[51] Chen ZP, Zeng WJ, Lei YM, Liang WB, Yang X, Yuan R, et al. Engineering of a multi-modular DNA nanodevice for spatioselective imaging and evaluation of NK cell-mediated cancer immunotherapy. Angew Chem Int Ed Engl. 2025;64(2):e202414064. 39375853 10.1002/anie.202414064

[52] Kulasinghe A, Monkman J, Shah ET, Matigian N, Adams MN, O’Byrne K. Spatial profiling identifies prognostic features of response to adjuvant therapy in triple negative breast cancer (TNBC). Front Oncol. 2021;11:798296. 35083152 10.3389/fonc.2021.798296 PMC8784863

[53] Yang P, Lu J, Zhang P, Zhang S. Comprehensive analysis of prognosis and immune landscapes based on lipid-metabolism- and ferroptosis-associated signature in uterine corpus endometrial carcinoma. Diagnostics. 2023. 10.3390/diagnostics13050870. 36900015 10.3390/diagnostics13050870 PMC10000778

[54] Ying L, Zhang C, Reuben A, Tian Y, Jin J, Wang C, et al. Immune-active tumor-adjacent tissues are associated with favorable prognosis in stage I lung squamous cell carcinoma. iScience. 2023;26(9):107732. 37694148 10.1016/j.isci.2023.107732 PMC10483046

[55] Huo Q, Ning L, Xie N. Identification of GZMA as a potential therapeutic target involved in immune infiltration in breast cancer by integrated bioinformatical analysis. Breast Cancer (Dove Medical Press). 2023;15:213–26. 36926265 10.2147/BCTT.S400808 PMC10013577

[56] Santiago L, Castro M, Sanz-Pamplona R, Garzón M, Ramirez-Labrada A, Tapia E, et al. Extracellular granzyme a promotes colorectal cancer development by enhancing gut inflammation. Cell Rep. 2020;32(1):107847. 32640217 10.1016/j.celrep.2020.107847

[57] Roufas C, Chasiotis D, Makris A, Efstathiades C, Dimopoulos C, Zaravinos A. The expression and prognostic impact of immune cytolytic activity-related markers in human malignancies: a comprehensive meta-analysis. Front Oncol. 2018;8:27. 29515971 10.3389/fonc.2018.00027 PMC5826382

