# Supplementary material for: A cross-scale multimodal framework identifies clinically actionable immunotherapy biomarkers in melanoma through bulk to single-cell and spatial transcriptomics integration
Source: Hum Genomics. 2026 Mar 16;20:77. doi: 10.1186/s40246-026-00919-w (PMC13104449; doi:10.1186/s40246-026-00919-w)
Supplement: Supplementary file 2 — Supplementary Material 2. [file 40246_2026_919_MOESM2_ESM.docx]

**A cross-scale multimodal framework identifies clinically actionable immunotherapy biomarkers in melanoma through bulk to single-cell and spatial transcriptomics integration**

Wuda Huoshen^1#^, Kun Yuan^2#^, Junkai Xiong^1#^, Yilong Lin^3#^, Wenjie Yu^4,5,6^, Yun Xie^7^, Qian Yuan^8^, Xinyue Zhang^9^, Changqing Dong^10^, Chen Sun^11*^, Sha Yi^12*^.

**Supplementary Figures**


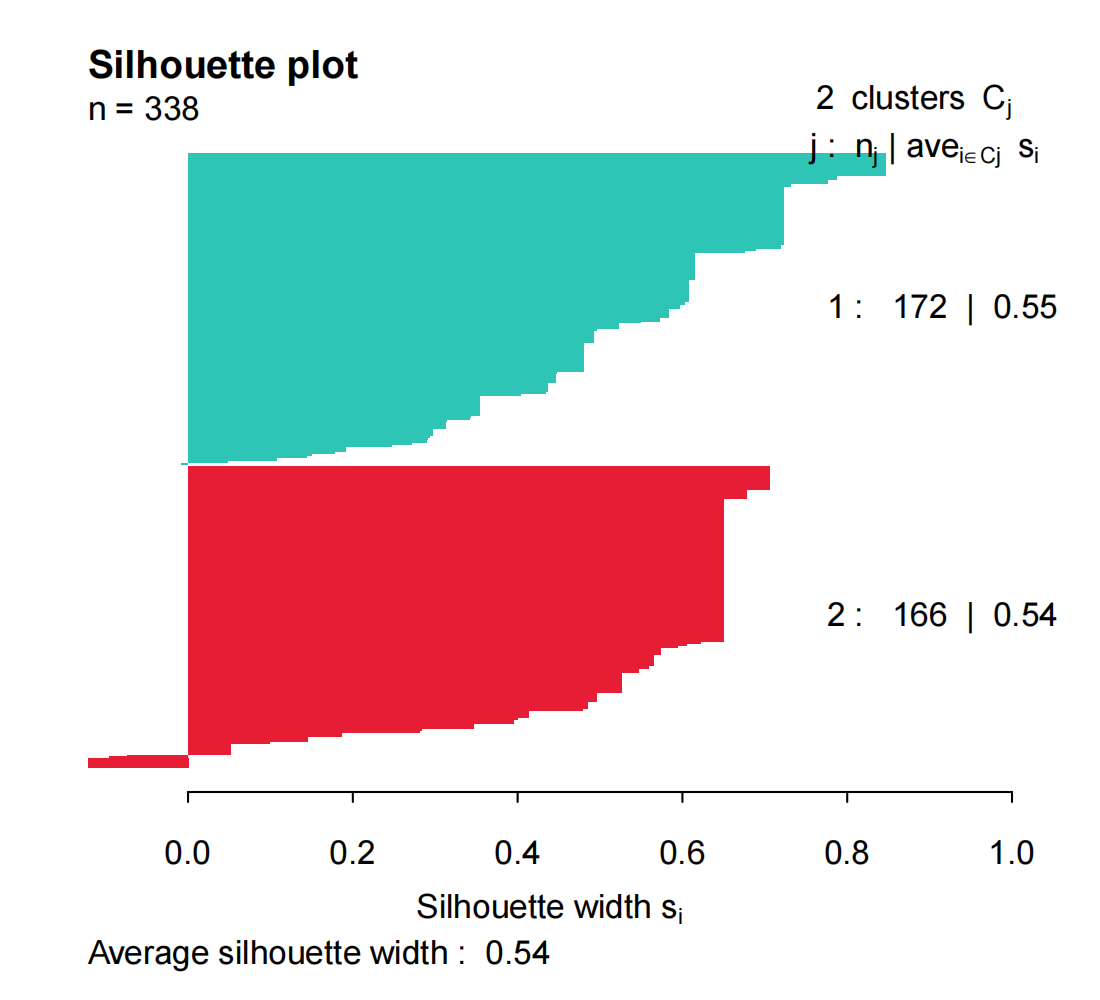


Figure S1. The sample similarity of each subgroup was assessed by calculating the Silhoutte score.


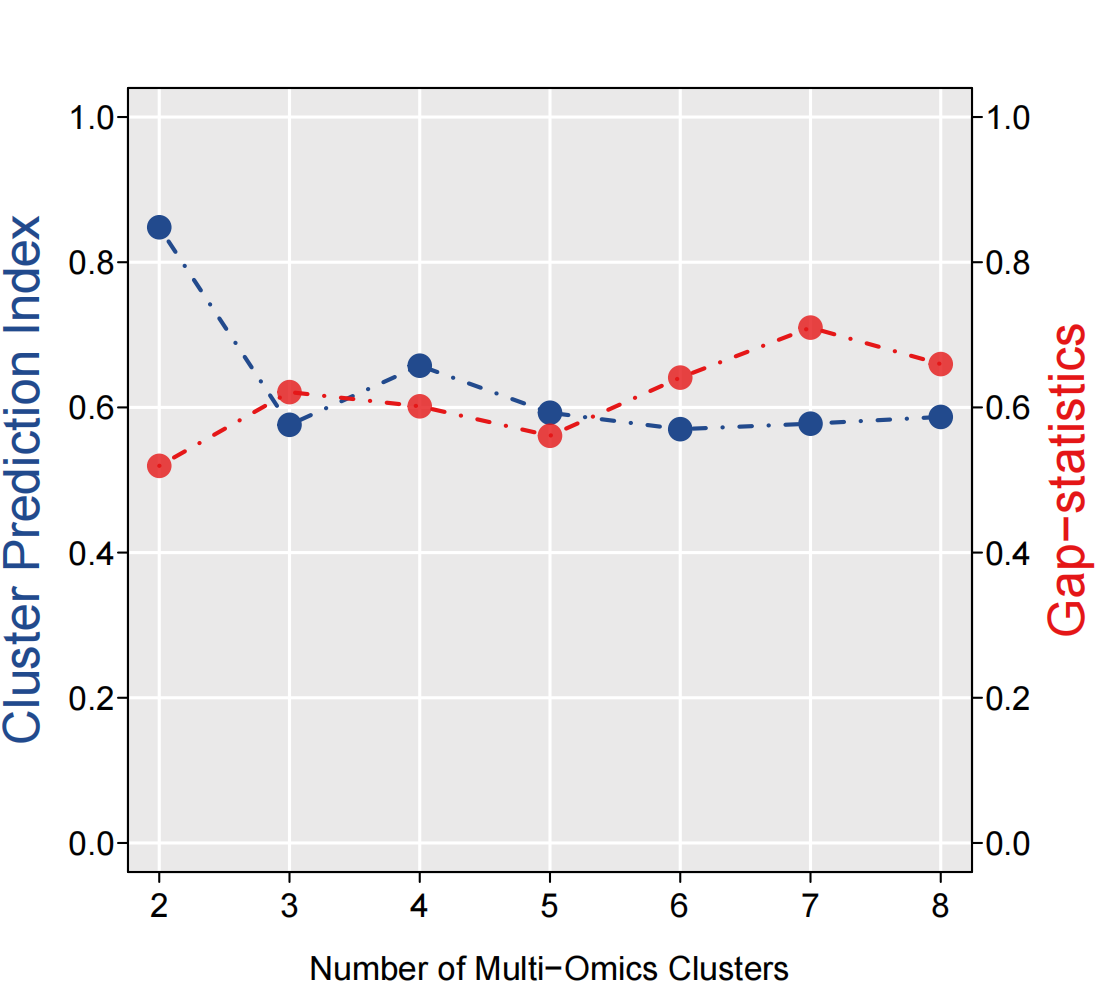


Figure S2. The cluster prediction index and gap statistical analysis of the multi-omics clusters.


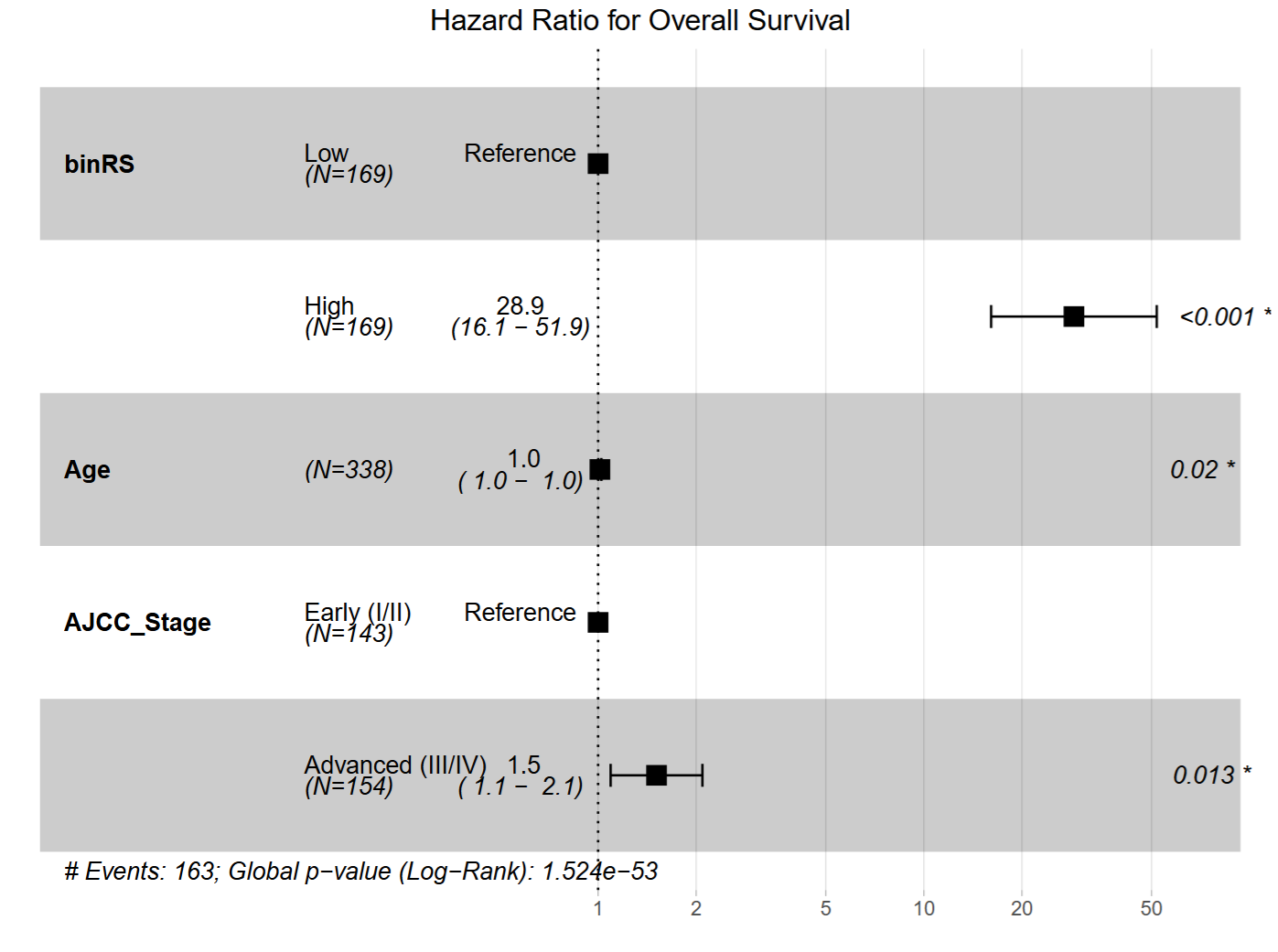


Figure S3. The single-gene expression analysis and OS survival analysis of SELL in meta-GEO cohorts.


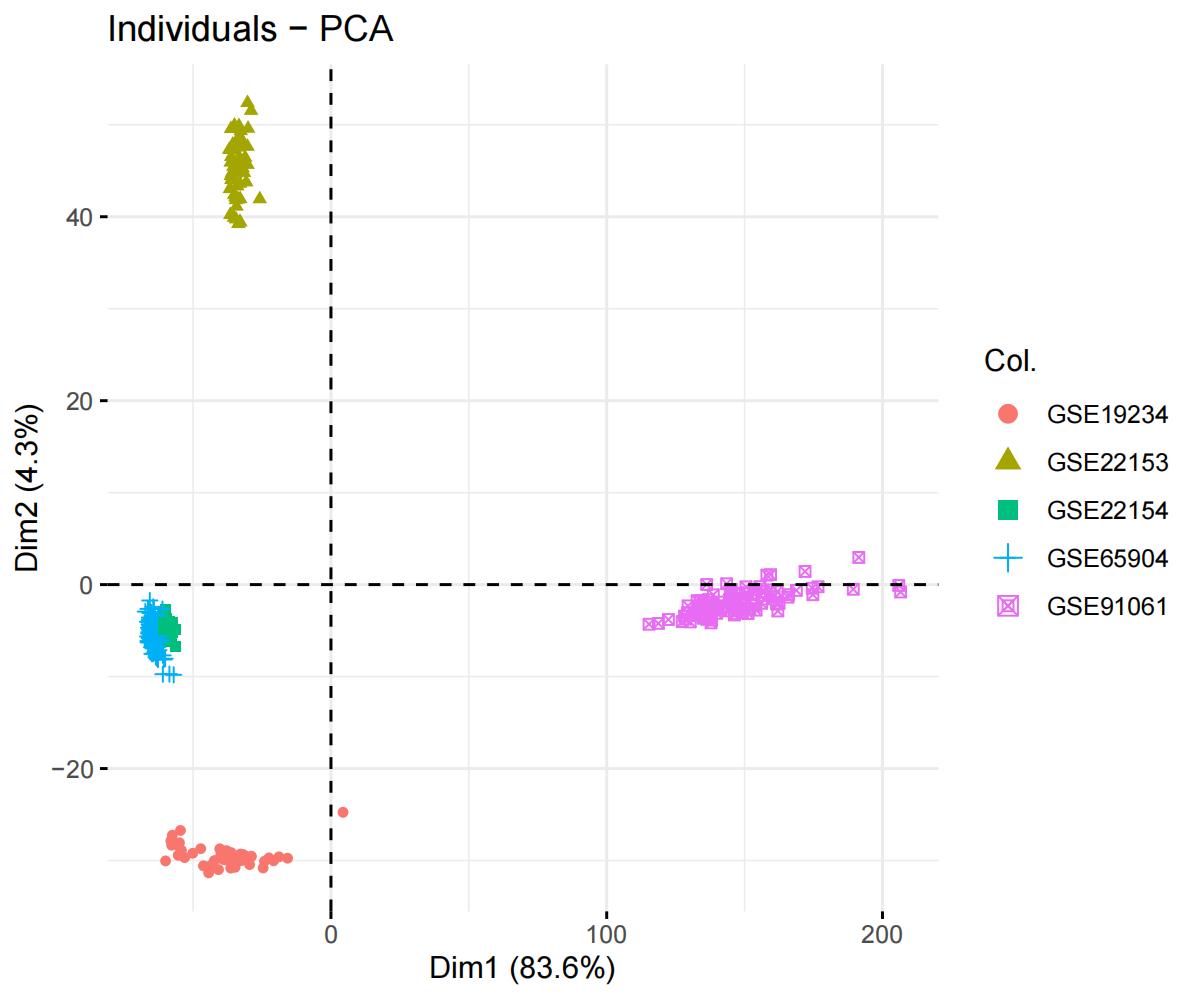


Figure S4. Principal component analysis (PCA) of samples before data merging.


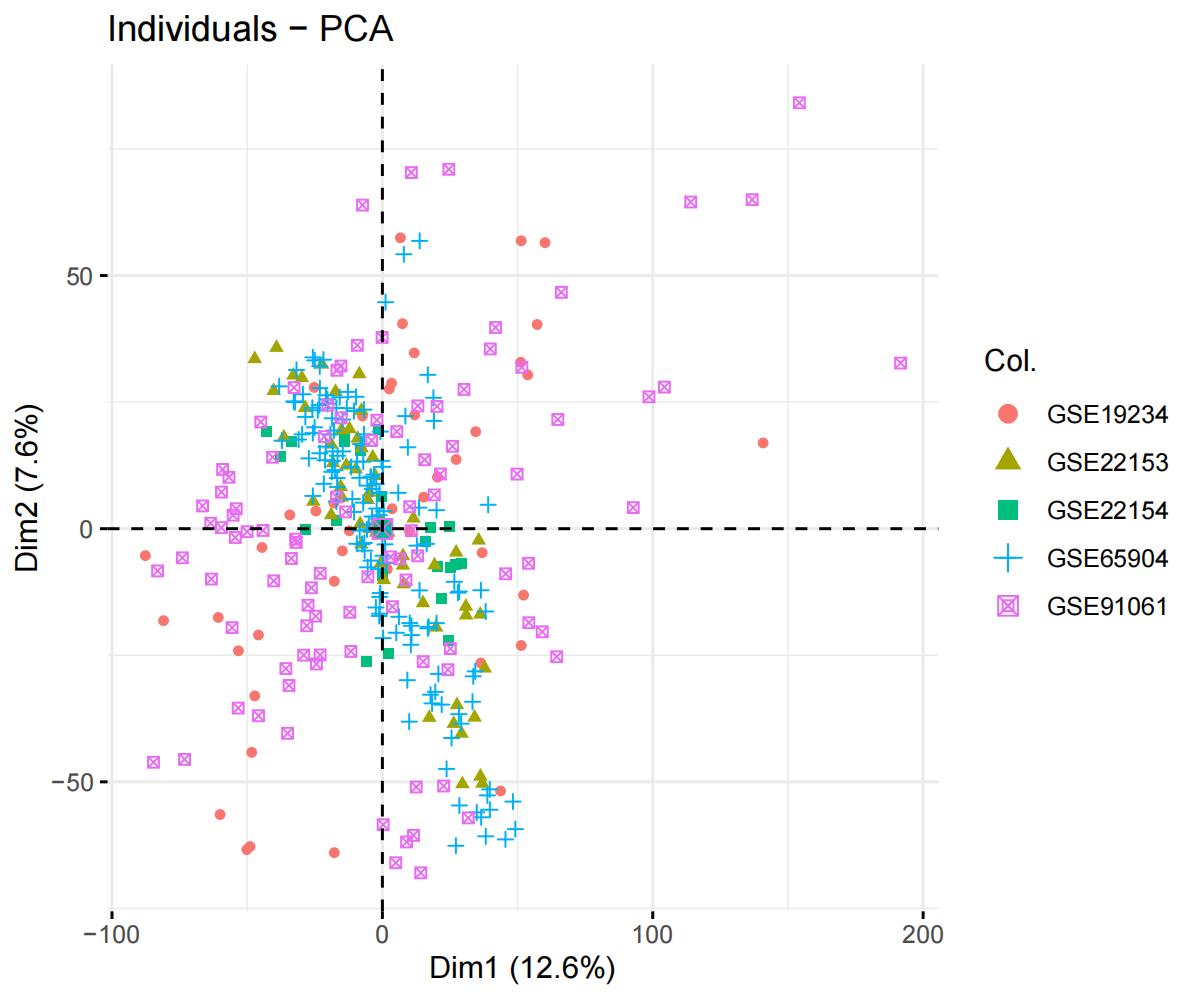


Figure S5. Principal component analysis (PCA) of samples after data merging.


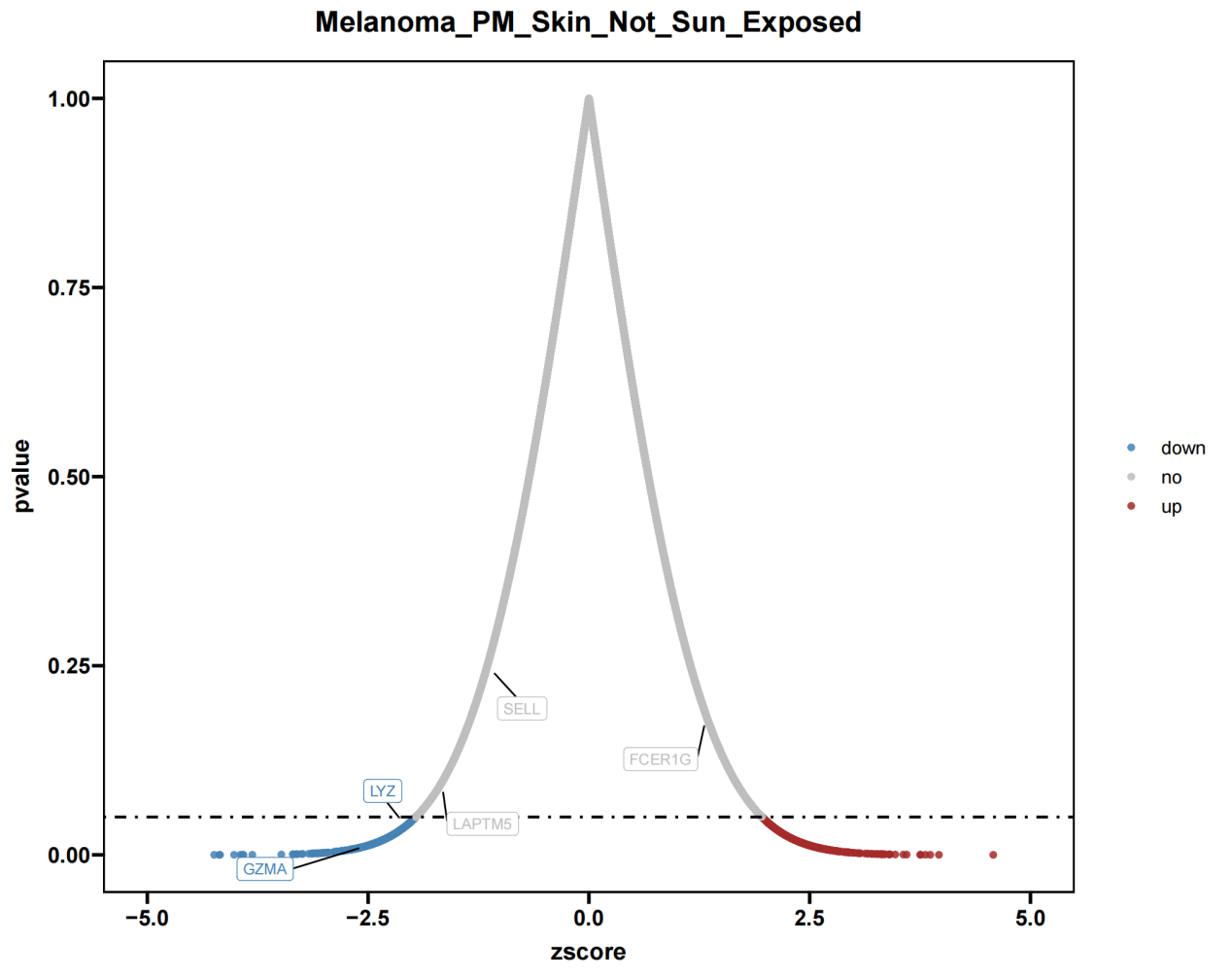


Table S6. The TWAS analysis validation results of TCGA and meta-GEO cohorts in Skin Not Sun Exposed Suprapubic.


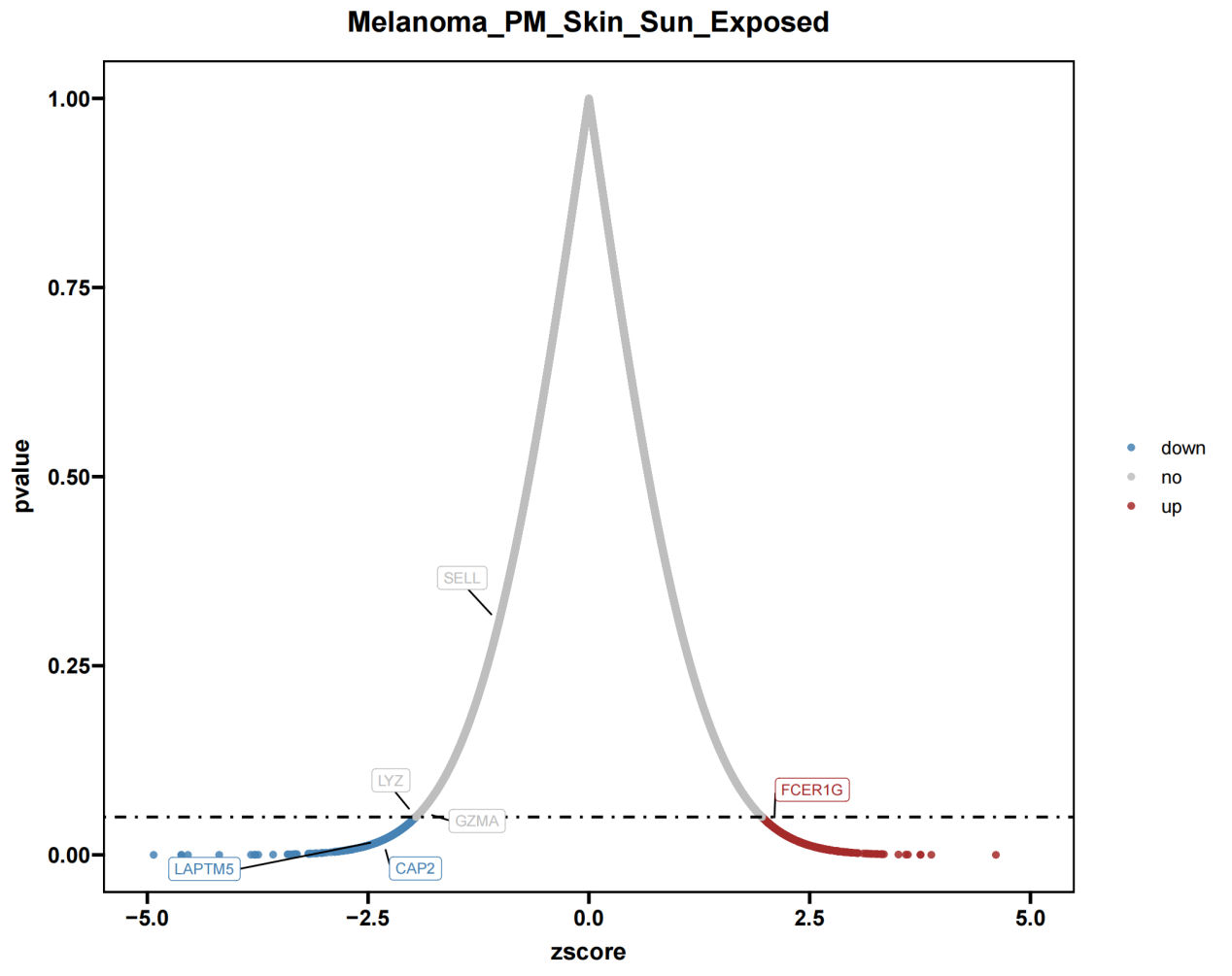


Table S7. The TWAS analysis validation results of TCGA and meta-GEO cohorts in Skin Sun Exposed Lower leg.


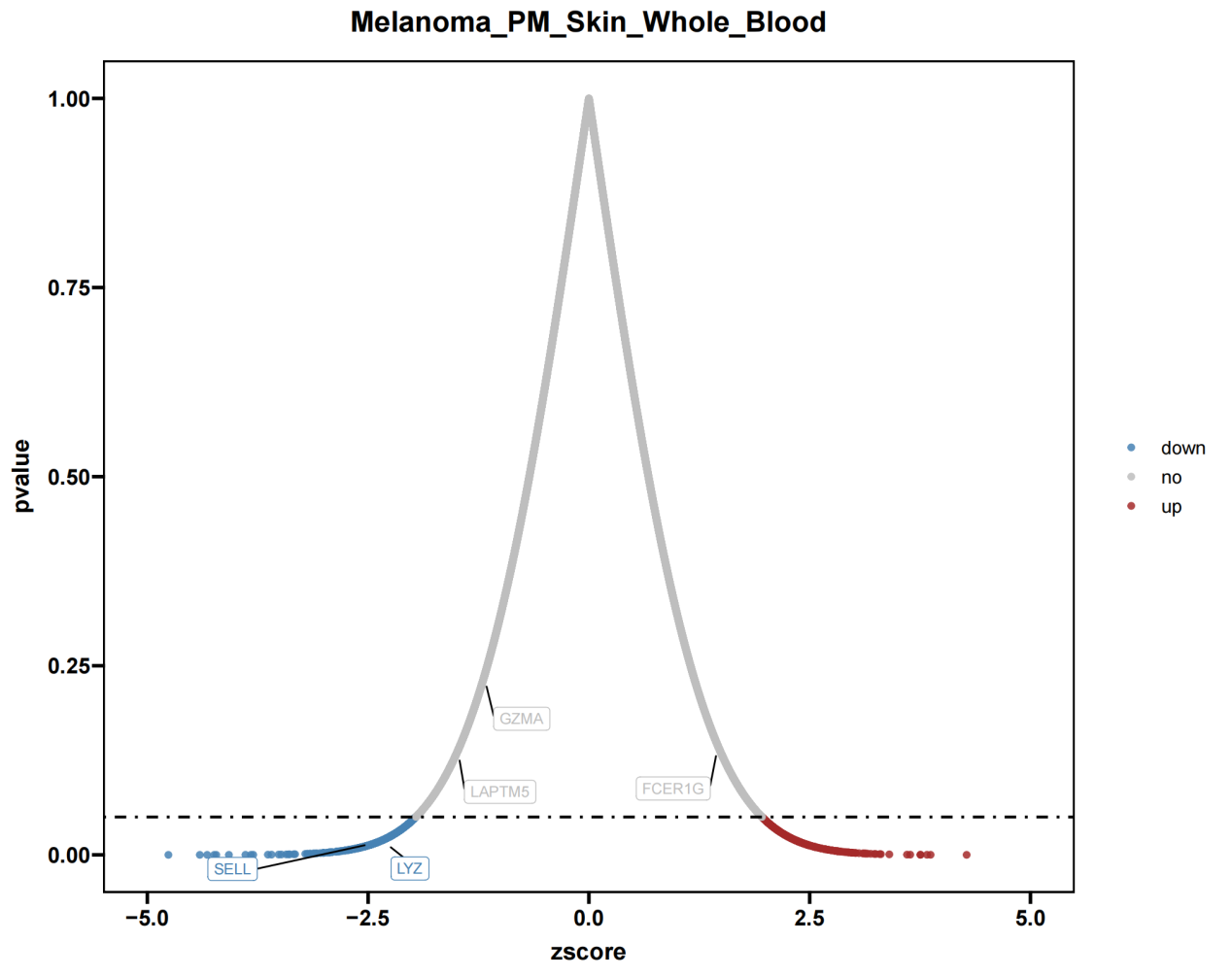


Table S8. The TWAS analysis validation results of TCGA and meta-GEO cohorts in Whole Blood.


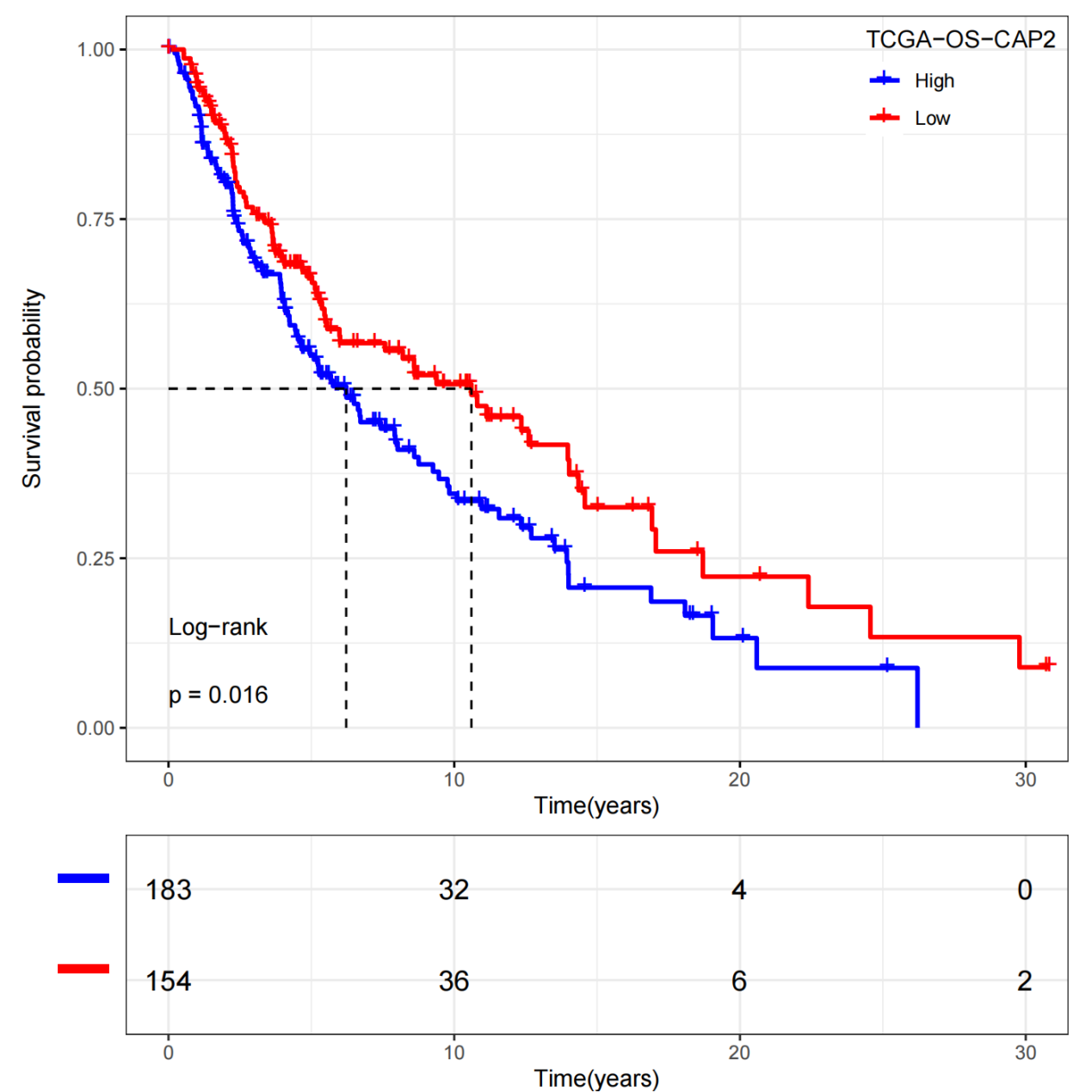


Figure S9. The single-gene expression analysis and OS survival analysis of CAP2 in TCGA cohorts.


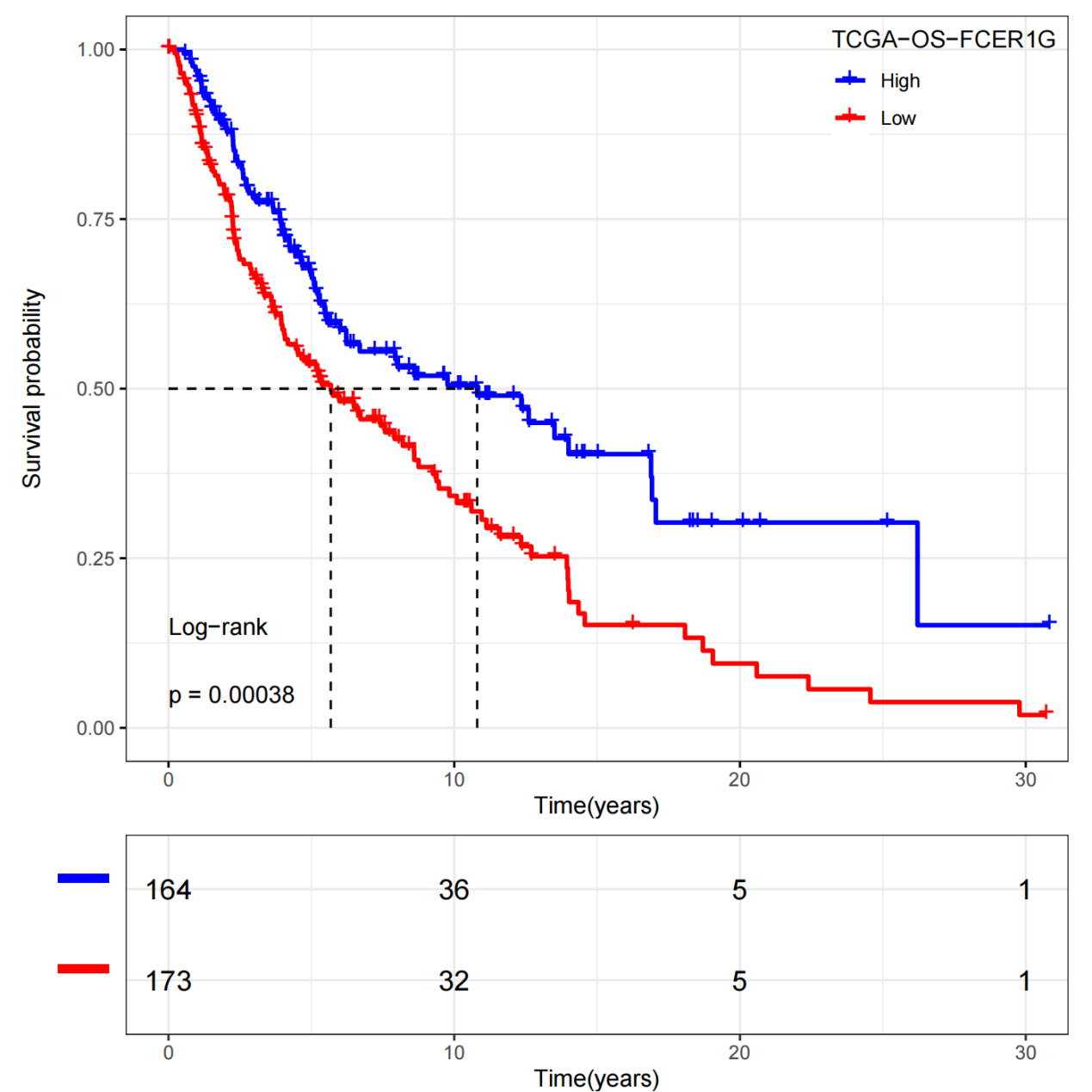


Figure S10. The single-gene expression analysis and OS survival analysis of FCER1G in TCGA cohorts.


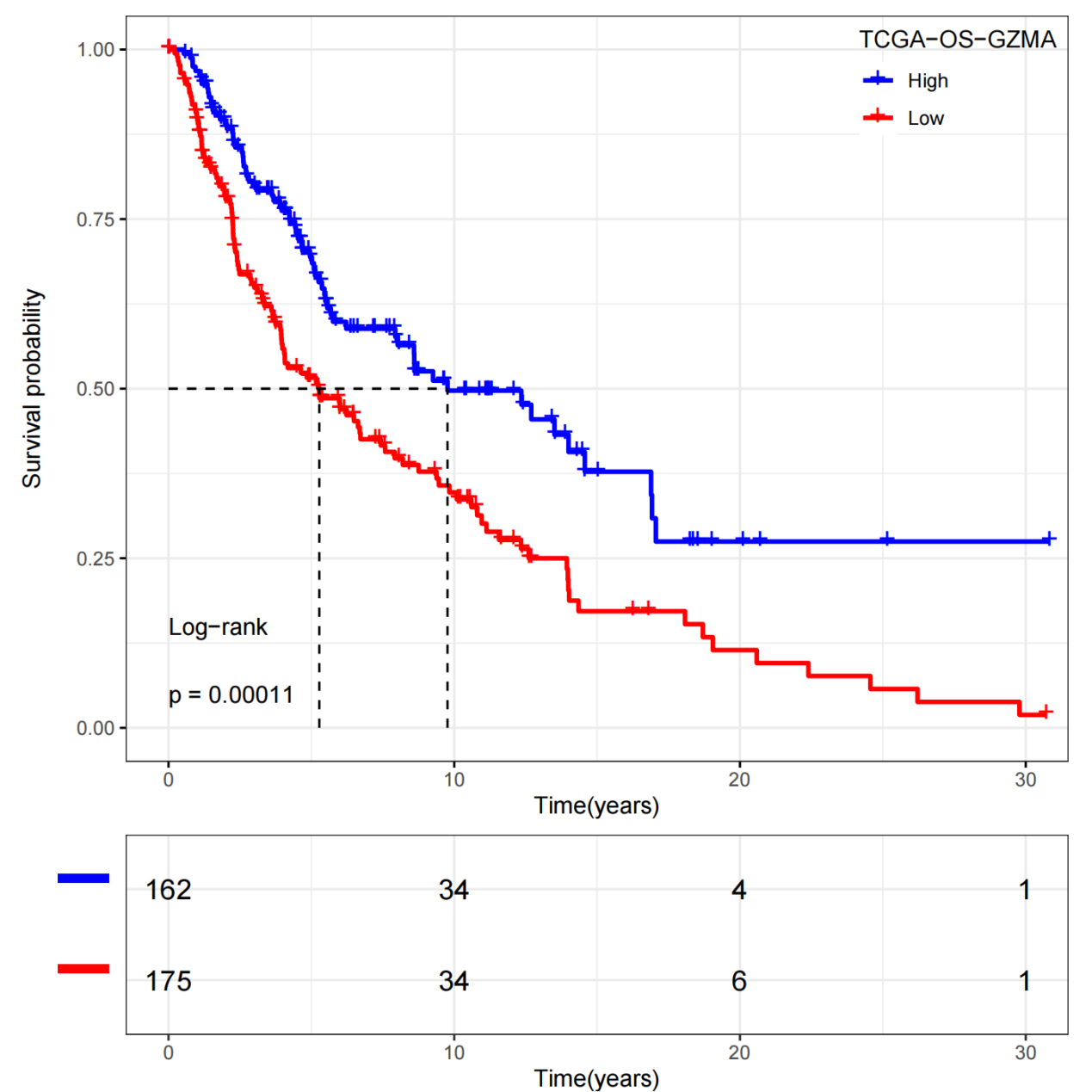


Figure S11. The single-gene expression analysis and OS survival analysis of GZMA in TCGA cohorts.


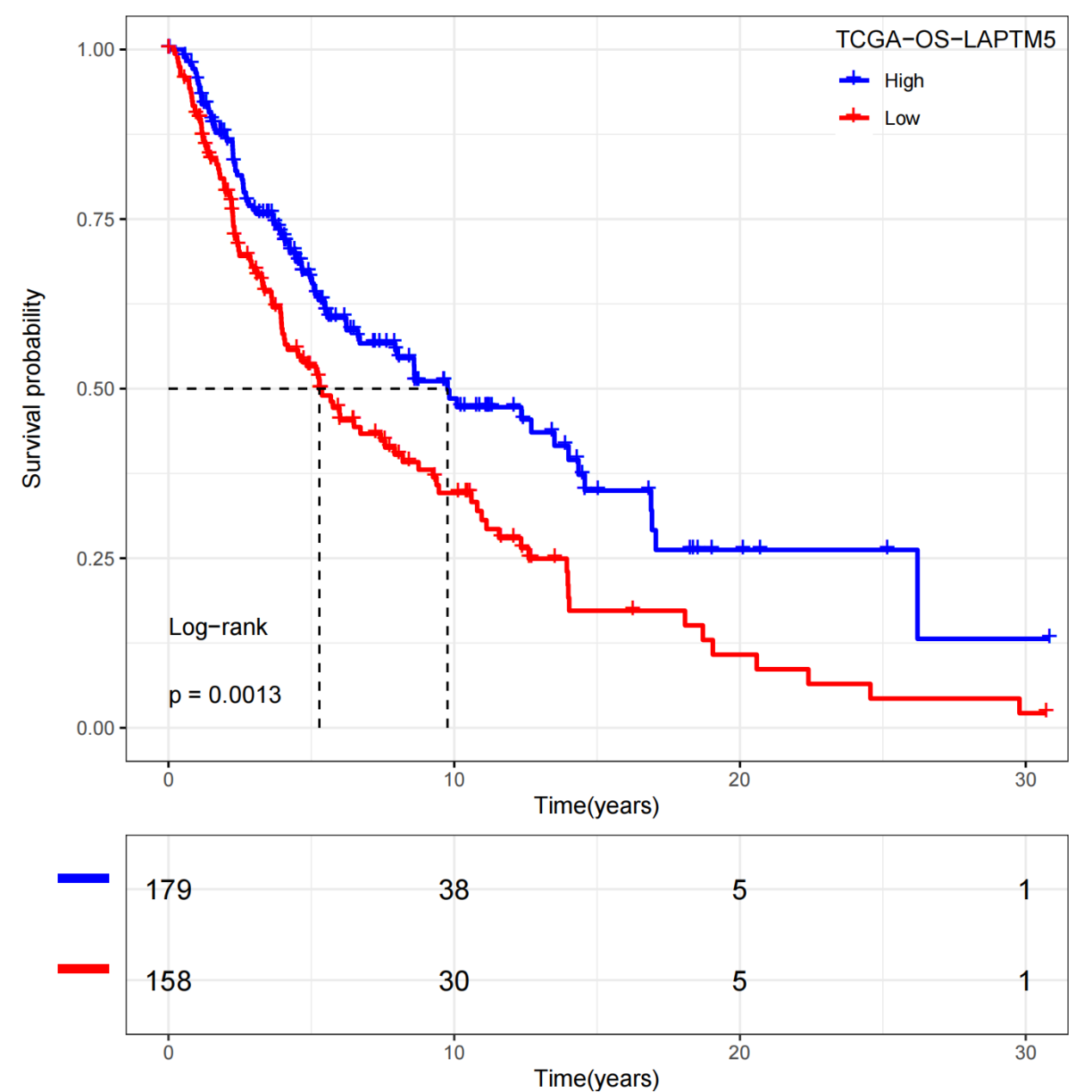


Figure S12. The single-gene expression analysis and OS survival analysis of LAPTM5 in TCGA cohorts.


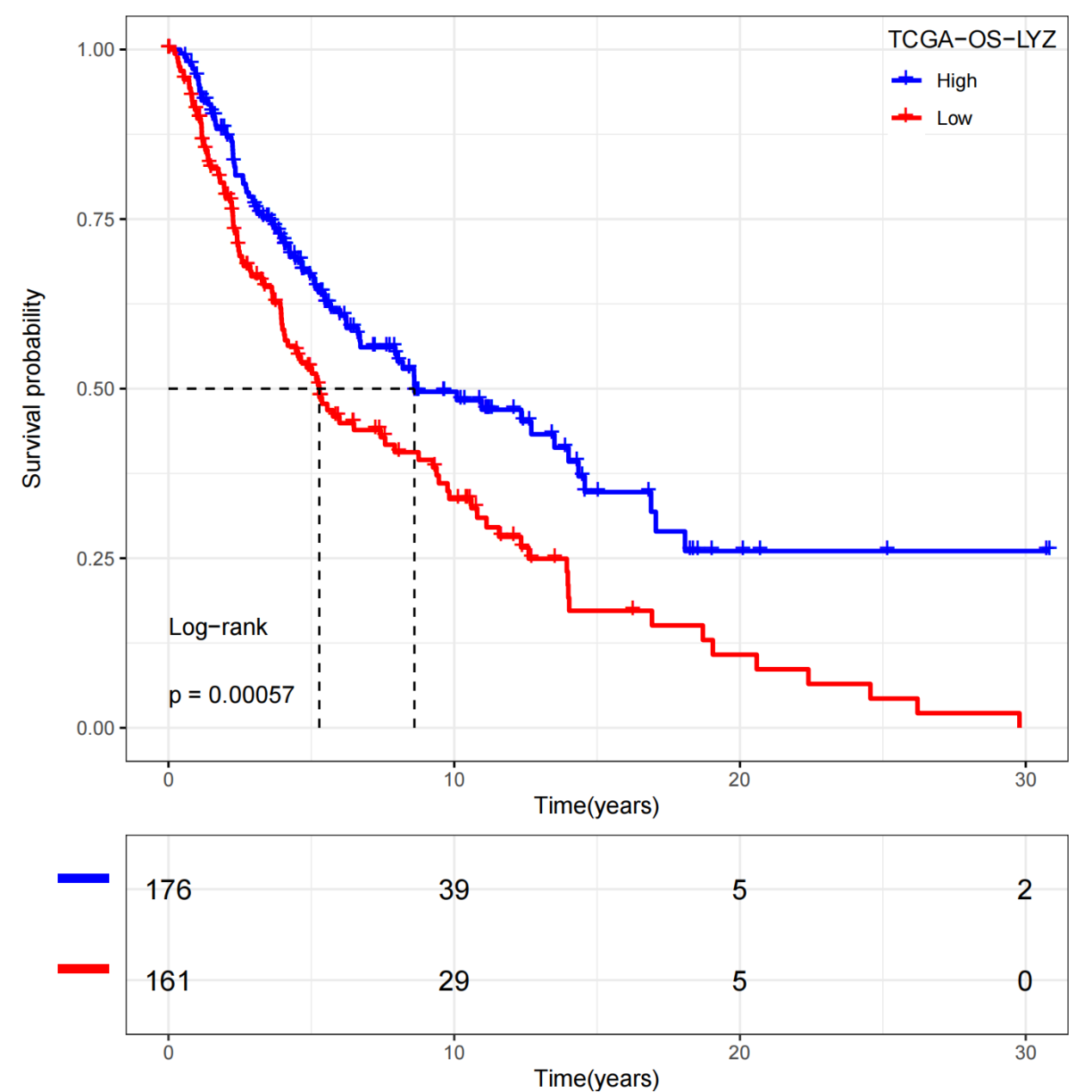
Figure S13. The single-gene expression analysis and OS survival analysis of LYZ in TCGA cohorts.


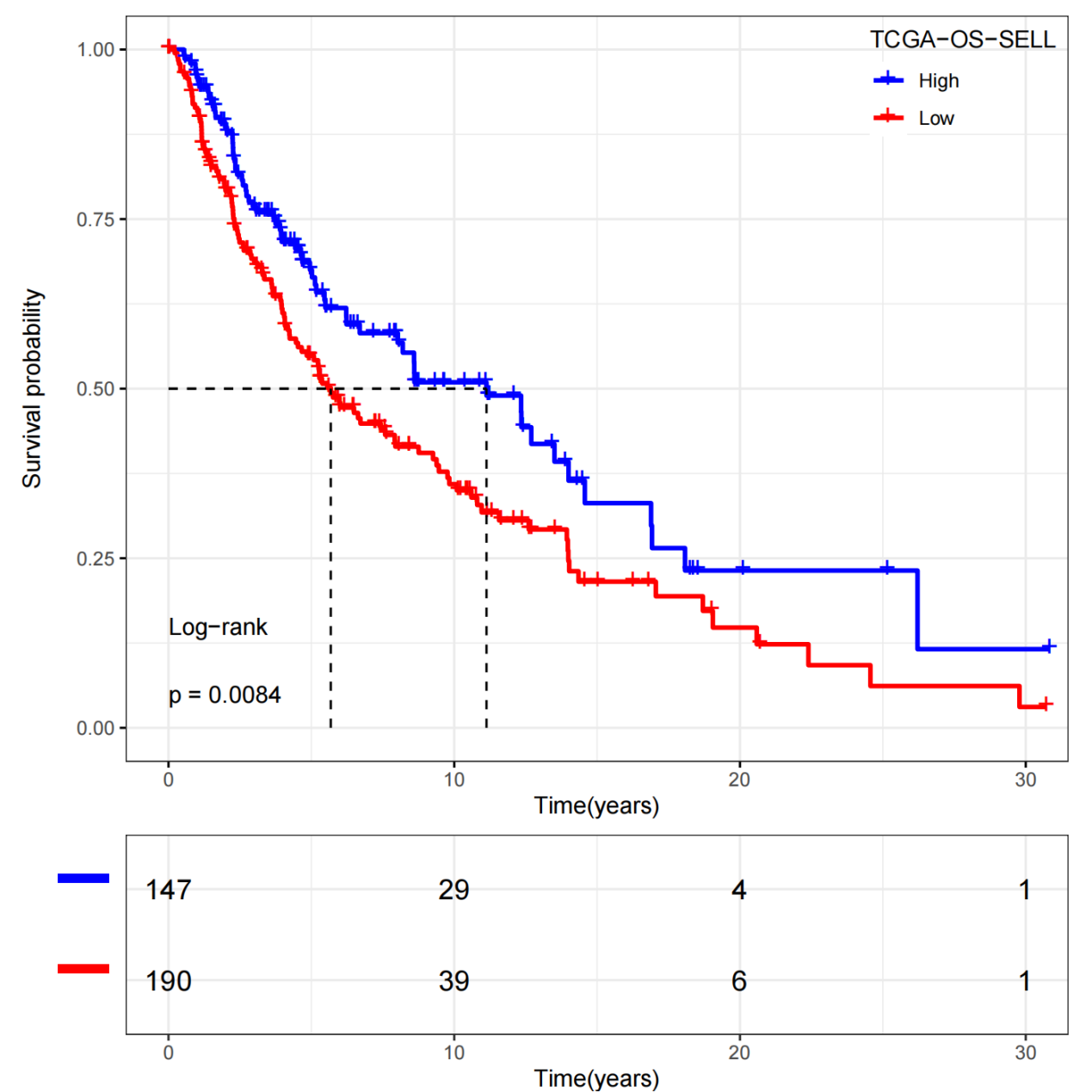


Figure S14. The single-gene expression analysis and OS survival analysis of SELL in TCGA cohorts.


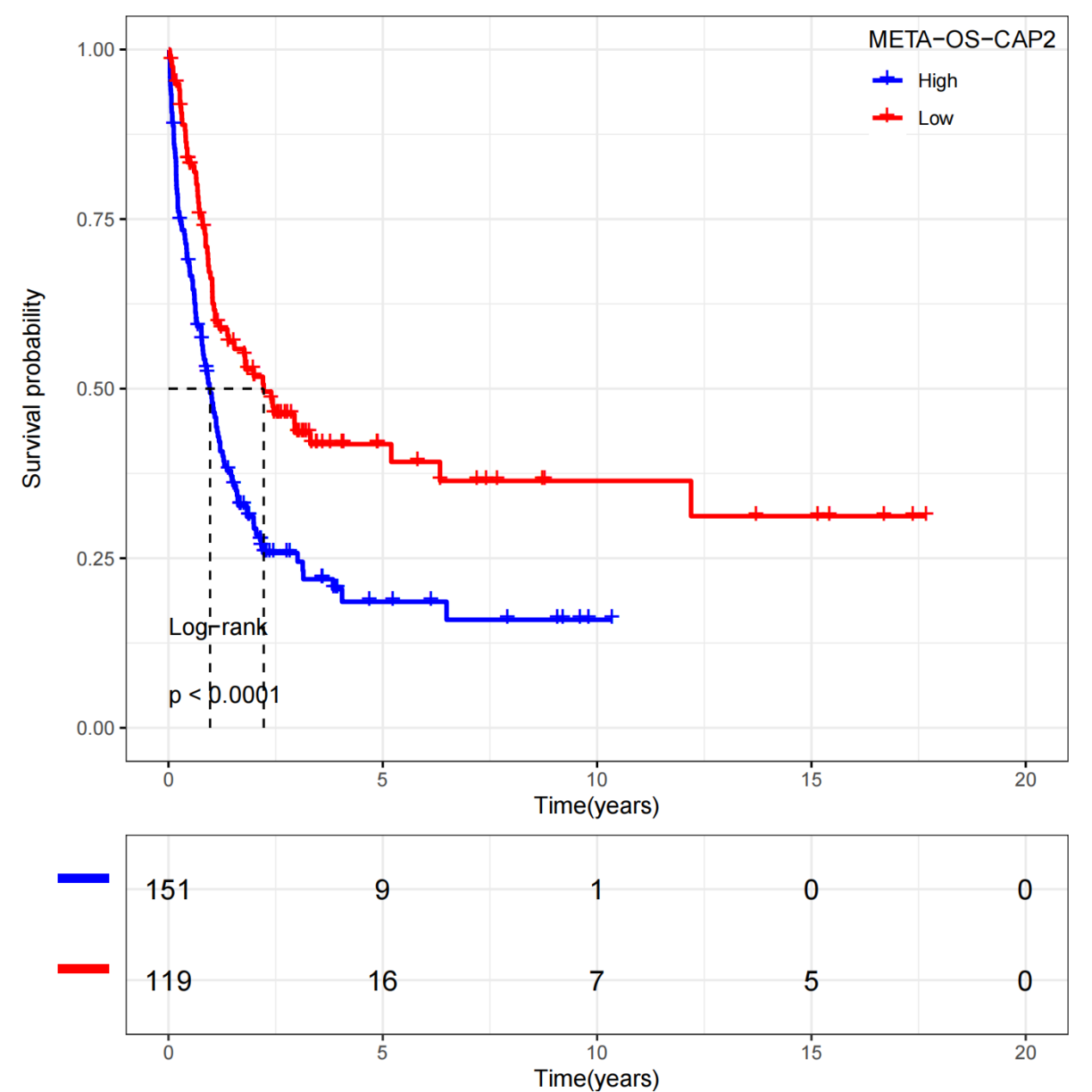
Figure S15. The single-gene expression analysis and OS survival analysis of CAP2 in meta-GEO cohorts.


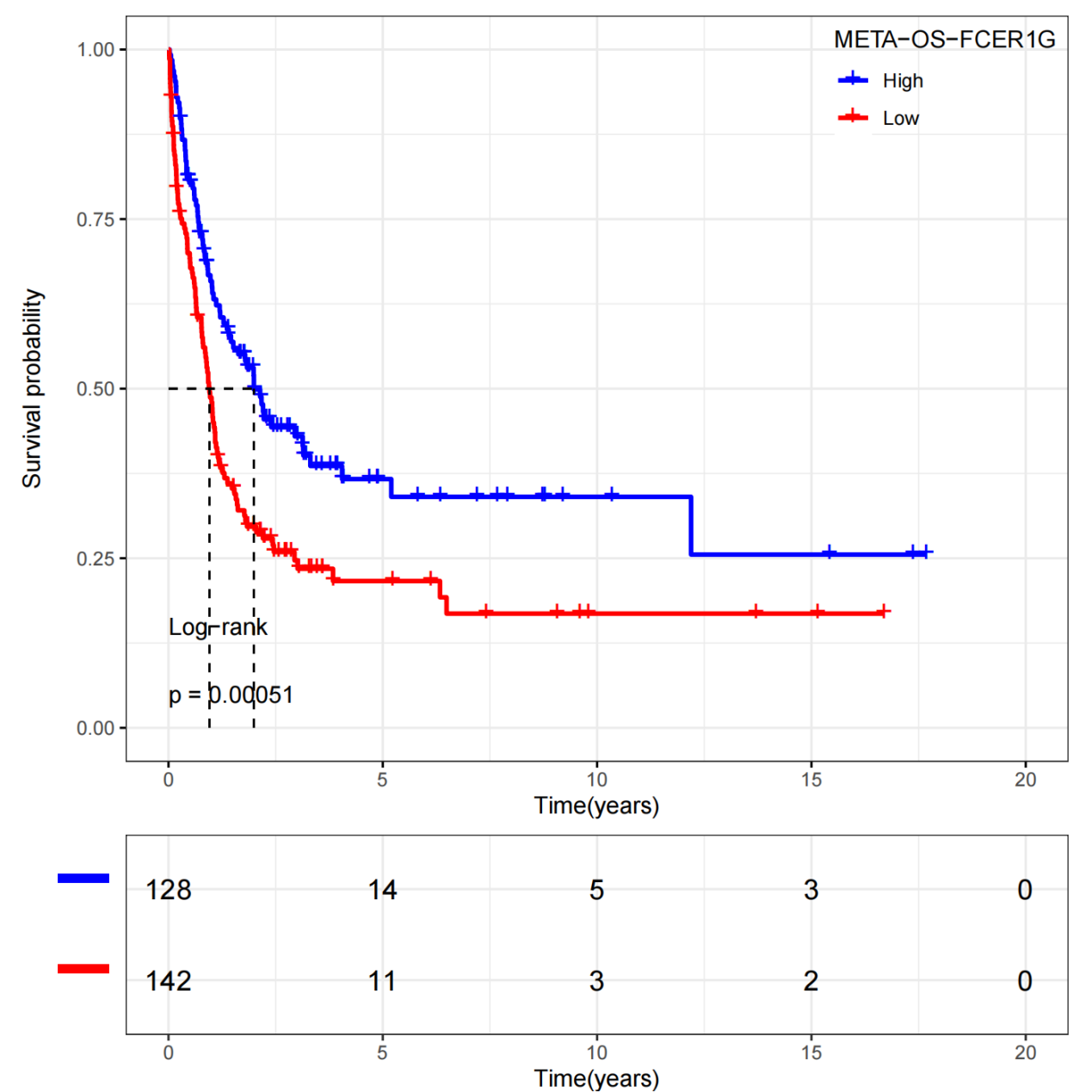
Figure S15. The single-gene expression analysis and OS survival analysis of FCER1G in meta-GEO cohorts.
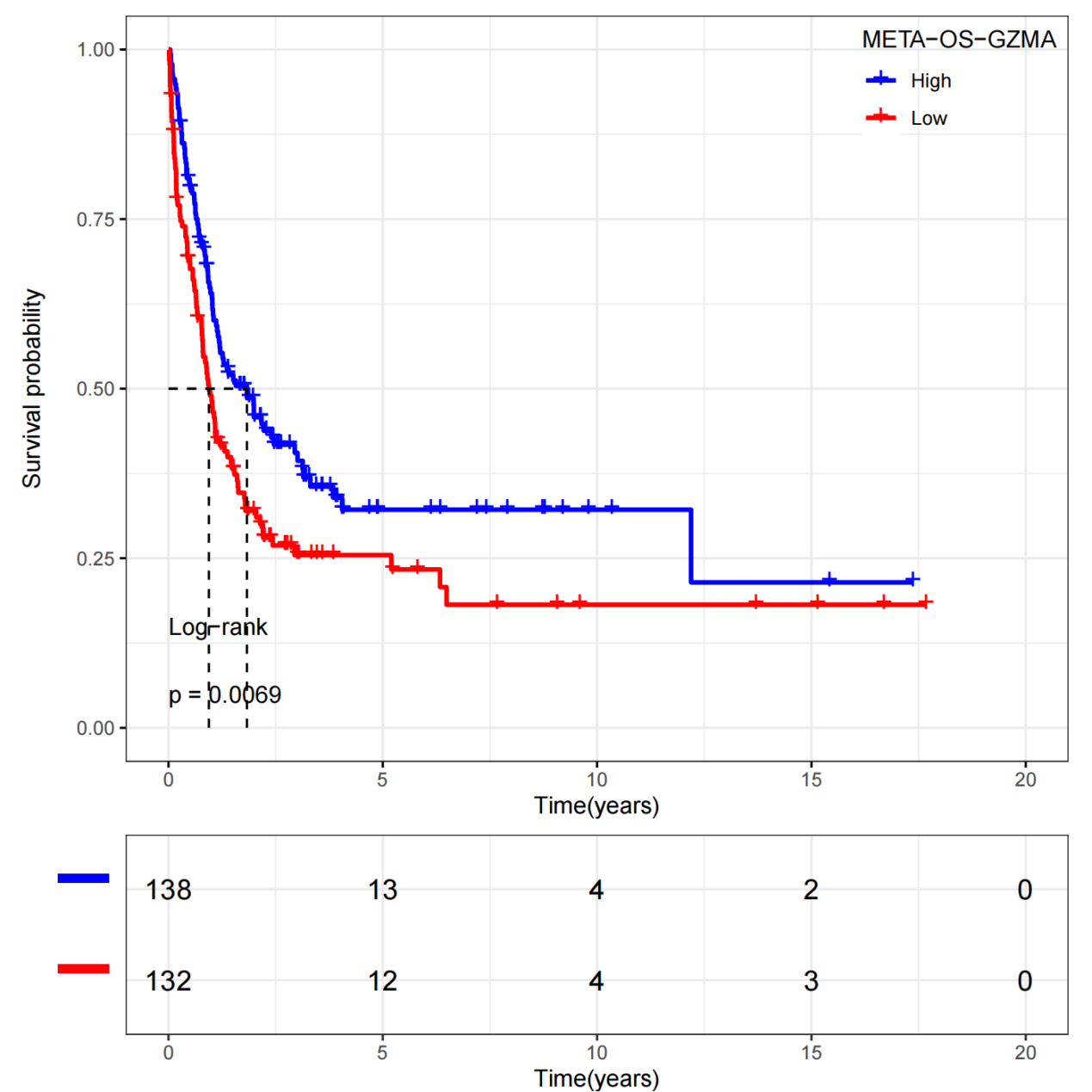
Figure S17. The single-gene expression analysis and OS survival analysis of GZMA in meta-GEO cohorts.
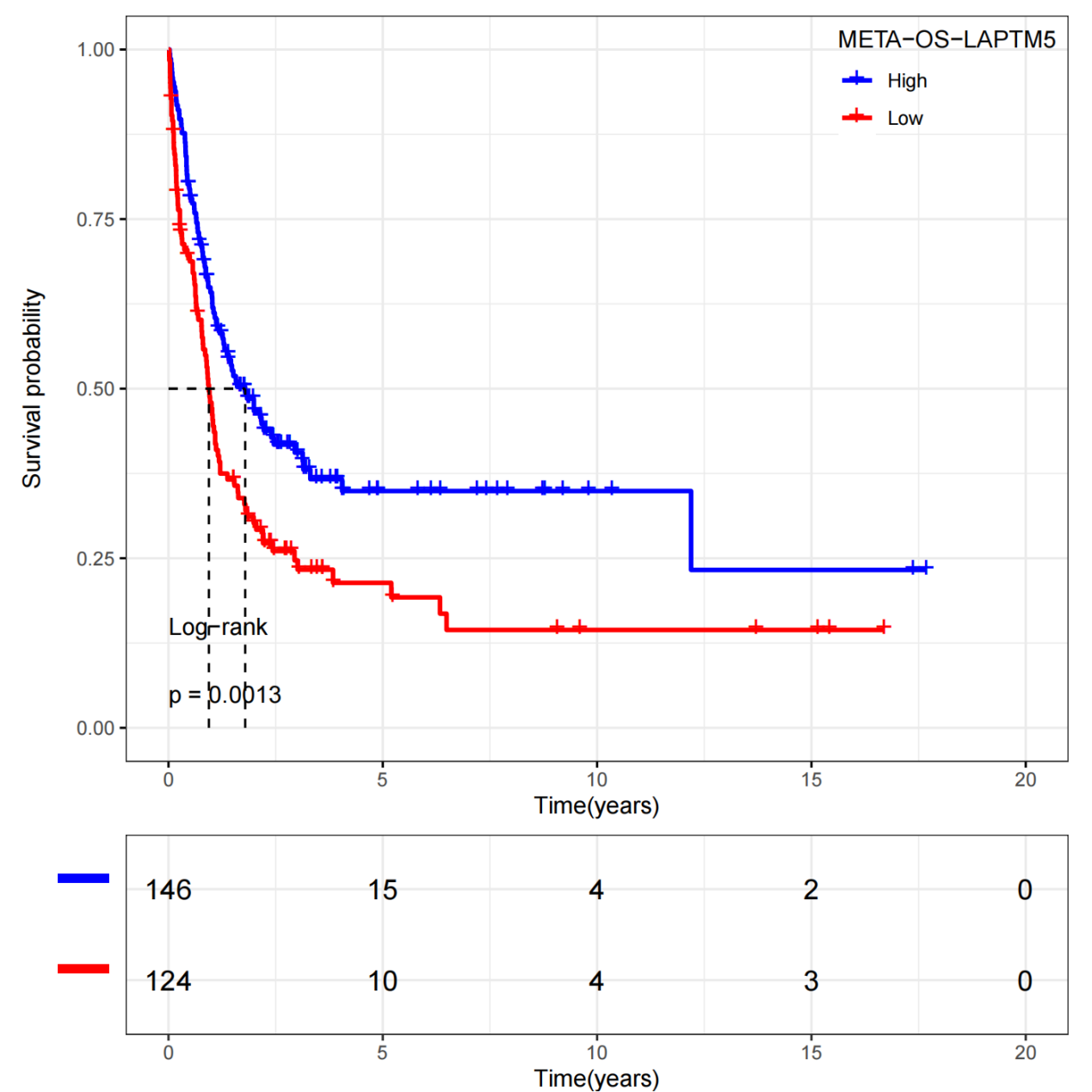


Figure S18. The single-gene expression analysis and OS survival analysis of LAPTM5 in meta-GEO cohorts.
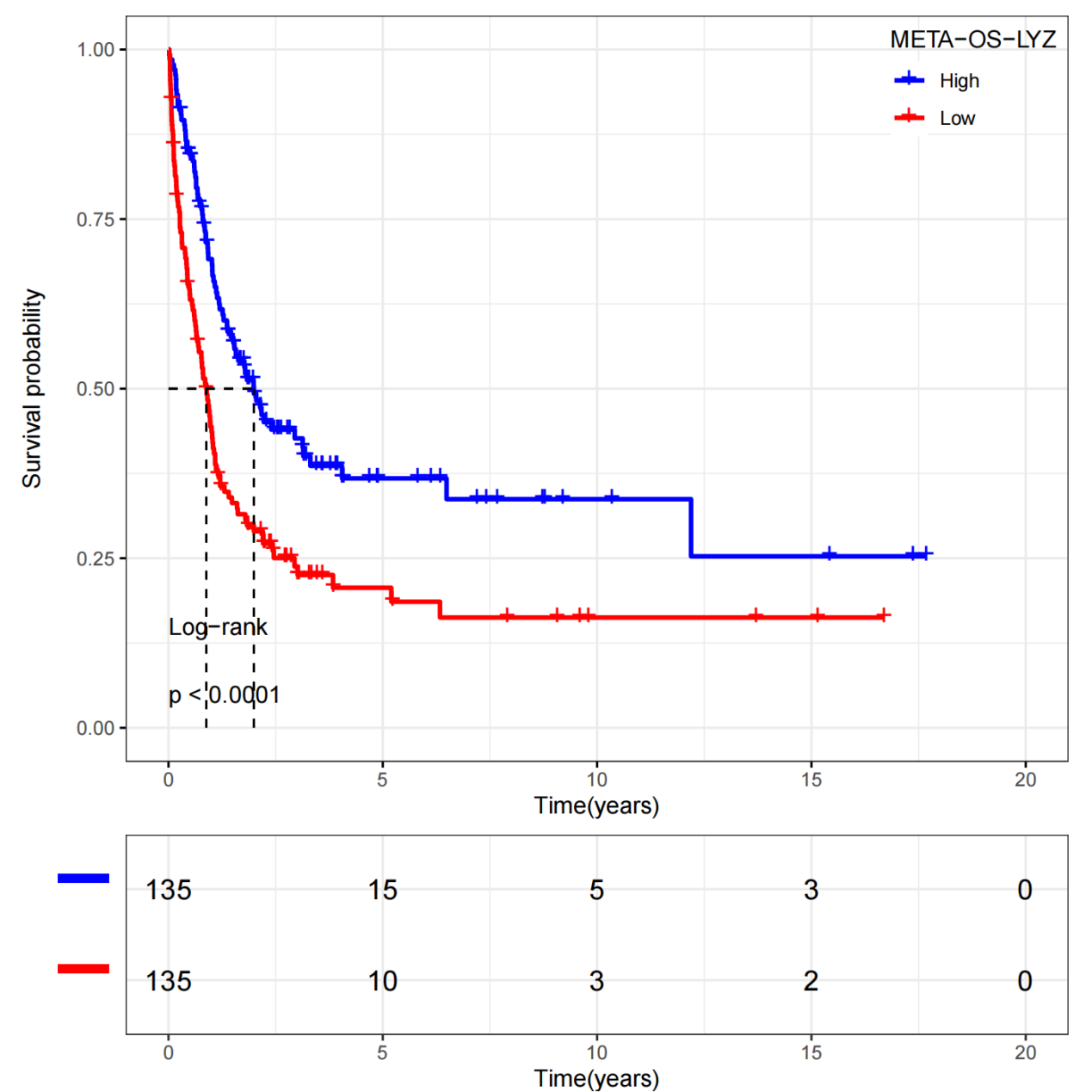
Figure S19. The single-gene expression analysis and OS survival analysis of LYZ in meta-GEO cohorts.
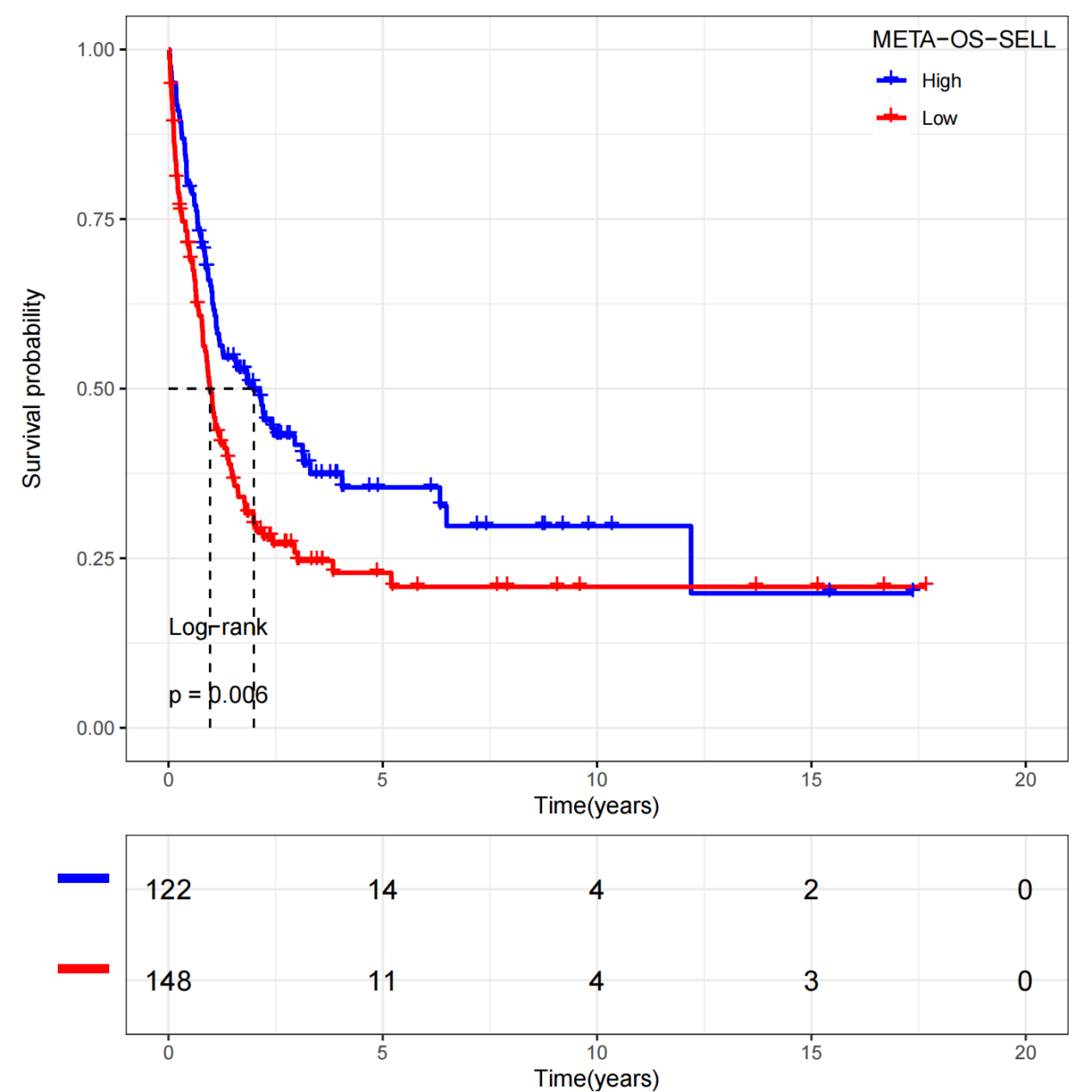


Figure S20. The single-gene expression analysis and OS survival analysis of SELL in meta-GEO cohorts.
